# Carmofur prevents cell cycle progression by reducing E2F8 transcription in temozolomide-resistant glioblastoma cells

**DOI:** 10.1038/s41420-023-01738-x

**Published:** 2023-12-12

**Authors:** Cyntanna C. Hawkins, Amber B. Jones, Emily R. Gordon, Yuvika Harsh, Julia K. Ziebro, Christopher D. Willey, Corinne Griguer, David K. Crossman, Sara J. Cooper, Sasanka Ramanadham, Ninh Doan, Anita B. Hjelmeland

**Affiliations:** 1 Department of Cell, Developmental and Integrative Biology, University of Alabama at Birmingham, Birmingham, AL USA; 2 HudsonAlpha Institute for Biotechnology, Huntsville, AL USA; 3 Graduate Biomedical Sciences, Division of Neuropathology, Department of Pathology, O’Neal Comprehensive Cancer Center, University of Alabama School of Medicine, Birmingham, AL USA; 4 Department of Radiation Oncology, Heersink School of Medicine, University of Alabama at Birmingham (UAB-SOM), Birmingham, AL USA; 5 Free Radical & Radiation Biology Program, Department of Radiation Oncology, The University of Iowa, Iowa City, IA USA; 6 Department of Genetics, University of Alabama at Birmingham, Birmingham, AL USA; 7 Comprehensive Diabetes Center, University of Alabama at Birmingham, Birmingham, AL USA; 8 Baptist South Medical Center, Montgomery, AL USA

**Keywords:** CNS cancer, Translational research

## Abstract

Sphingolipid metabolism is dysregulated in many cancers, allowing cells to evade apoptosis through increased sphingosine-1-phosphate (S1P) and decreased ceramides. Ceramidases hydrolyze ceramides to sphingosine, which is phosphorylated by sphingosine kinases to generate S1P. The S1P allows cells to evade apoptosis by shifting the equilibrium away from ceramides, which favor cell death. One tumor type that exhibits a shift in the sphingolipid balance towards S1P is glioblastoma (GBM), a highly aggressive brain tumor. GBMs almost always recur despite surgical resection, radiotherapy, and chemotherapy with temozolomide (TMZ). Understanding sphingolipid metabolism in GBM is still limited, and currently, there are no approved treatments to target dysregulation of sphingolipid metabolism in GBM. Carmofur, a derivative of 5-fluorouracil, inhibits acid ceramidase (ASAH1), a key enzyme in the production of S1P, and is in use outside the USA to treat colorectal cancer. We find that the mRNA for *ASAH1*, but not other ceramidases, is elevated in recurrent GBM. When TMZ-resistant GBM cells were treated with carmofur, decreased cell growth and increased apoptosis were observed along with cell cycle perturbations. RNA-sequencing identified decreases in cell cycle control pathways that were specific to TMZ-resistant cells. Furthermore, the transcription factor and G1 to S phase regulator, E2F8, was upregulated in TMZ-resistant versus parental GBM cells and inhibited by carmofur treatment in TMZ-resistant GBM cells, specifically. These data suggest a possible role for E2F8 as a mediator of carmofur effects in the context of TMZ resistance. These data suggest the potential utility of normalizing the sphingolipid balance in the context of recurrent GBM.

## Introduction

The balance between ceramides and sphingosine-1-phosphate (S1P), termed the sphingolipid rheostat, is shifted toward S1P to promote malignancy of many cancers [1]. Ceramidases (acid, neutral, and alkaline) are responsible for the hydrolysis of ceramides to sphingosine, which is then phosphorylated by sphingosine kinases to S1P [2]. Inhibiting ASAH1 in colon cancer using carmofur sensitized cells to oxaliplatin, a platinum-based chemotherapy [3]. Similarly, head and neck cancer cells overexpressing ASAH1 have enhanced resistance to cisplatin, another platinum-based chemotherapy [4]. Consistently, ASAH1 is upregulated in prostate cancer cell lines as well as in human tissue following radiation. Applying that understanding to prostate cancer PDXs, ASAH1 inhibition sensitized cells to radiation and prevented recurrence [5]. When the activities of the different ceramidases were analyzed, ASAH1, specifically, was found to be upregulated following radiation. Doxorubicin, another chemotherapeutic drug, elevated ceramides in melanoma cells and ablation of ASAH1 using CRISPR-Cas9 amplified apoptosis in response to doxorubicin. ASAH1-null cells were also unable to recover and develop resistance to doxorubicin compared to wildtype cells [6]. Together, these studies emphasize ASAH1 roles in therapeutic resistance for a variety of cancers.

Glioblastoma (GBM) is a malignant brain tumor which almost always recurs despite aggressive treatment with surgical resection, radiation, and chemotherapy with temozolomide (TMZ). ASAH1 is overexpressed in glioma tissue from patients compared to normal brain, while the other ceramidases remain unchanged [7, 8]. ASAH1 has also been shown to increase after radiotherapy in GBM, potentially due to the induction of ceramides caused by radiation [9]. One approach that many laboratories, including ours, have utilized is the identification of repurposed inhibitors. The benefit of using repurposed drugs is the existing years of clinical data showing efficacy and potential side effects in other disease indications. For this reason, we chose to determine if we could repurpose carmofur for the treatment of GBM, including TMZ-resistant GBM. Carmofur, a derivative of 5-fluorouracil, was found to inhibit ASAH1 and increase ceramides both in vitro and in vivo, specifically in the brains of mice [10].

Our laboratory previously described a role for ASAH1 in the migration of GBM cells [8]. As part of this study, we noted a growth inhibitory effect of carmofur at seven days and used the calculated IC50 values for the shorter-term migration assays [8]. The cells used in these experiments were from primary GBMs that had not been exposed to temozolomide (TMZ), the standard of care chemotherapy for GBM. While we and others demonstrated a growth inhibitory effect with carmofur in GBM cells, its ability to affect TMZ-resistant GBM has not been determined [11, 12]. We therefore sought to understand whether ASAH1 could remain a target in recurrent GBM and/or have a role in resistance to chemotherapy, specifically TMZ. As GBM cells rapidly become resistant to TMZ treatment, there is a critical need for therapies that increase the efficacy of TMZ or remain effective in recurrent GBM [13]. Currently, promoter methylation of the DNA repair enzyme O[6]-methylguanine-DNA methyltransferase (MGMT) is the only biomarker for TMZ response [14], and it often dictates enrollment in clinical trials [15]. Here, we report that carmofur increases apoptosis and senescence in TMZ-resistant GBM cells through downregulation of the cell cycle pathway, correlating with expression of E2F8, a cell cycle regulator.

## Results

### TMZ-resistant GBM cells remain sensitive to carmofur

**Fig. 1:**
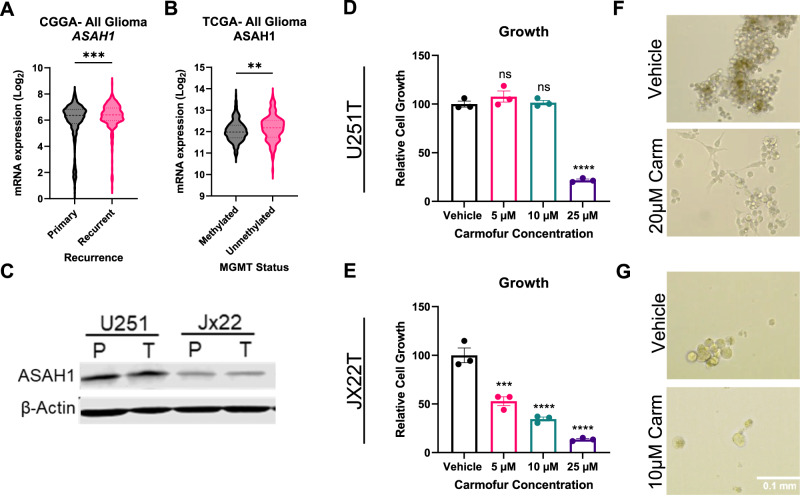
TMZ-resistant GBM cells maintain sensitivity to pharmacologic inhibition of ASAH1. *ASAH1* expression is higher in recurrent glioma patients. mRNA expression for *ASAH1* in primary and recurrent glioma patients was accessed using GlioVis (http://gliovis.bioinfo.cnio.es; accessed on 28 December 2021). **A** Comparisons were made between recurrent and primary glioma patients for *ASAH1* (*n* = 651 for primary, *n* = 333 for recurrent). Data were analyzed using the non-parametric, Mann-Whitney t-test. **B** Comparison of gene expression for methylated and unmethylated gliomas in TCGA dataset for *ASAH1* (*n* = 477 for methylated, *n* = 161 for unmethylated). Data were analyzed using the non-parametric, Mann-Whitney t-test. **C** ASAH1 is expressed in TMZ-resistant cells as assessed by immunoblot analysis. **D**, **E** Carmofur decreased cell growth in two TMZ-resistant GBM cell types as assessed by Cell Titer Glo after 7 days. **D** U251T data were analyzed with one-way ANOVA and Dunnett’s multiple comparisons test for comparison to vehicle control (*n* = 3). **E** JX22T data were analyzed with Kruskal-Wallis test and the uncorrected Dunn’s test for multiple comparisons for comparison to vehicle control (*n* = 3). **F**, **G** Representative images of U251T and JX22T cells 7 days following treatment with carmofur. Data are shown as mean ± SEM. ** for *p* < 0.05, *** for *p* < 0.002, **** for *p* < 0.0001.

We recently reported that *ASAH1* expression was associated with decreased median survival in GBM patients [8]. Based on our understanding of ASAH1 in primary gliomas and the association of ASAH1 with chemoresistance in other tumors, we sought to determine if ceramidases were elevated in recurrent gliomas. Ceramidases are identified according to their optimum pH for activity and further designated based on their cellular localization [2] (Fig. S1A, B). When we assessed the expression of available ceramidases (*ACER1* was unavailable) in primary and recurrent gliomas using the Chinese Glioma Genome Atlas (CGGA) [16], we found that only *ASAH1* was increased in recurrent gliomas while the other ceramidases were unchanged (Fig. 1A, S1C–E). We used the data from the CGGA for this analysis, because the CGGA has the largest number of recurrent patient samples. We also sought to determine whether ceramidase expression correlated with MGMT promoter methylation status, because MGMT promoter-unmethylated gliomas are more resistant to TMZ [17]. Using The Cancer Genome Atlas (TCGA) [18], we found that *ASAH1* was elevated in MGMT unmethylated gliomas, but the other ceramidases were unchanged (Fig. 1B, S1F–I). We used the data from the TCGA because the CGGA did not permit rapid assessment of MGMT promoter methylation status. The datasets were used for different analyses as the TCGA dataset only has 16 samples for recurrent GBM, and the CGGA dataset does not have promoter methylation data readily available through GlioVis.

Based on the high expression of *ASAH1* mRNA in glioma patients, we sought to determine if TMZ-resistant GBM cells are sensitive to carmofur, a pharmacologic inhibitor of ASAH1. We utilized two different TMZ-resistant models to assess growth. U251T were generated by serially treating the U251 cell line in vitro until cells were resistant to TMZ [19]. JX22T were generated by serially treating the patient-derived xenograft (PDX) JX22P with TMZ in vivo [20]. ASAH1 protein expression remained consistent in the TMZ-resistant GBM cells making it a relevant target in this population (Fig. 1C). Both TMZ-resistant cell types also remained sensitive to carmofur: TMZ-resistant GBM cell growth decreased with increasing concentrations of carmofur (Fig. 1D, E). The estimated IC50 for U251T and JX22T for future experiments were 20 µM and 10 µM, respectively. Consistent with the changes in growth assessed by Cell Titer Glo, we also observed decreases in cell number when TMZ-resistant GBM cells were treated with the IC50 of carmofur for each cell type (Fig. 1F, G).

### Carmofur treatment induces cell cycle changes and apoptosis in TMZ-resistant GBM cells

**Fig. 2:**
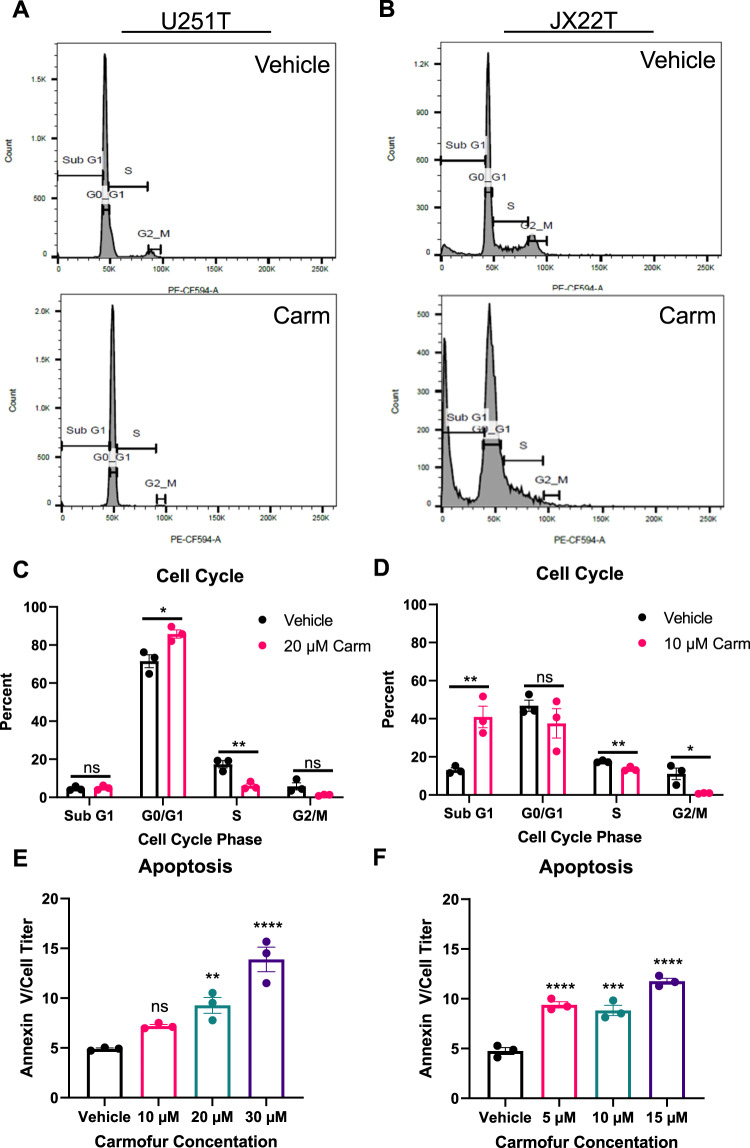
Carmofur shifts the cell cycle of TMZ-resistant cells to G0/G1 or sub-G0. **A**, **B** Representative flow cytometry histograms for propidium iodide (PI) staining in U251T and JX22T following 48 h of carmofur treatment with their respective IC50s. **C**, **D** Quantification of the percentage of cells in each cell cycle phase (*n* = 3 independent experiments analyzed by independent t test or the Mann-Whitney test for each cell cycle phase). **E**, **F** Carmofur treatment induced apoptosis in two different TMZ-resistant GBM cell types after 48 h as assessed by a non-lytic Annexin V/Necrosis assay (*n* = 3 analyzed by one-way ANOVA with Dunnett’s multiple comparisons test for comparison to vehicle control. Data are shown as mean ± SEM. * for *p* < 0.05, ** for *p* < 0.01, *** for *p* < 0.001, **** for *p* < 0.0001.

Upon observing changes in growth of TMZ-resistant GBM cells with carmofur treatment, we next determined if there were changes in the cell cycle. We treated U251T and JX22T with their respective carmofur IC50s and assessed cell cycle using propidium iodide after 48 hours. Both cell types displayed visual cell cycle shifts toward G0/G1 or sub G1 phases by flow cytometry (Fig. 2A, B). Quantification of the peaks corresponding to each cell cycle phase were graphed and comparisons made between the carmofur-treated and vehicle-treated cells. U251T cells showed increases in the percentage of cells in the G0/G1 phase and decreases in the percentage of cells in the S phase with the carmofur treatment (Fig. 2C). The JX22T cells displayed a dramatic increase in the percentage of cells in the Sub G1 phase suggesting apoptosis or necrosis (Fig. 2D). JX22T cells also had a decrease in the percentage of cells in both the S phase and the G2/M phase but did not have a change in G0/G1 likely due to the shift of cells to the Sub G0 phase overall. Next, we assessed apoptosis using a non-lytic Annexin V assay [21]. That analysis showed increased apoptosis with the carmofur treatment in both U251T and JX22T cells when normalized for cell number using Cell Titer Glo (Fig. 2E, F). Together, these data indicate that TMZ-resistant GBM cells have decreased growth in association with cell cycle shifts and increased apoptosis.

### Pathways related to cell cycle control are downregulated in TMZ-resistant GBM cells with carmofur treatment

**Fig. 3:**
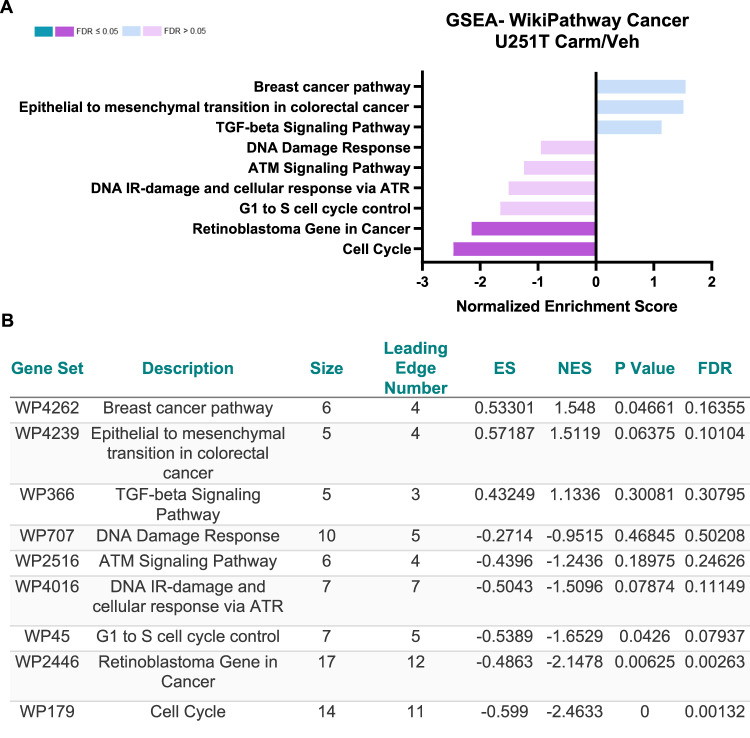
Carmofur treatment of TMZ-resistant GBM cells decreased pathways associated with cell cycle and DNA-repair. After 48 h of vehicle control (DMSO) or 20 µM carmofur, U251T cells were collected for RNA-sequencing (*n* = 3). **A** Gene set enrichment analysis (GSEA) was conducted for genes with a log2 FC > 1.5 or <-1.5 using Webgestalt (http://www.webgestalt.org; accessed on 20 October 2021). Wikipathway cancer functional database and a minimum of 5 genes/pathway were used for the analysis. Selection criteria for input genes included base mean >10 and a *p*-value < 0.05. **B** Differentially expressed pathways with their enrichment score (ES), normalized enrichment score (NES), *p* value, and false discovery rate (FDR).

To further understand the mechanism by which carmofur limits cell cycle progression and induces apoptosis, cells were analyzed by RNA-sequencing similar to our recent published study with U251P cells [8]. We identified 603 differentially expressed genes (DEGs) and selected genes with a base mean of 10, log_2_ fold change of >1.5 or <-1.5, and *p* value < 0.05 for gene set enrichment analysis (GSEA) using Webgestalt [22]. Differentially expressed pathways as determined by GSEA demonstrated significant decreases in cell cycle and retinoblastoma genes in cancer pathways (Fig. 3A, B). Interestingly, there were also trending decreases in pathways associated with the DNA damage response and DNA repair pathways. Similar pathway alterations were identified using Ingenuity Pathway Analysis (Figs. S2–3**)**.

### Carmofur decreases E2F8 mRNA expression specific to TMZ-resistant cells via transcriptional repression

**Fig. 4:**
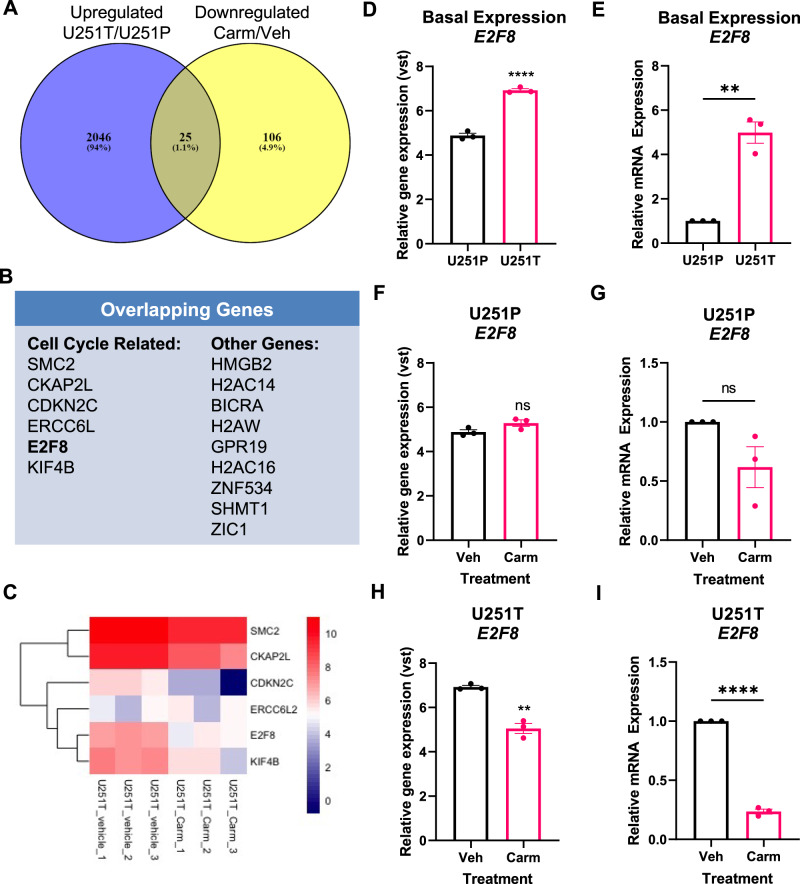
Identification of *E2F8* as a gene upregulated in TMZ-resistant versus parental U251 cells that is decreased by carmofur. **A** Genes upregulated in the U251T cells in comparison to parental cells were further compared with the genes downregulated by the carmofur treatment in the U251T cells. Unique pathways modulated by carmofur in TMZ-resistant cells were visualized using Venny 2.1 (https://bioinfogp.cnb.csic.es/tools/venny/). **B** 25 genes that are both upregulated in TMZ-resistant cells and downregulated by carmofur are shown. *E2F8*, a target of interest, is in bold. **C** Heatmap showing mRNA expression of cell cycle genes in U251T. mRNA expression for *E2F8* from **D** RNA-sequencing and **E** RT-qPCR in U251T compared to U251P basally. *E2F8* expression in **F** RNA-sequencing and **G** RT-qPCR for U251P cells treated with carmofur. *E2F8* expression in **H** RNA-sequencing and by **I** RT-qPCR for U251T treated with carmofur (*n* = 3 independent experiments analyzed by independent t test). Data are shown as mean ± SEM. ** for *p* < 0.01, ****p < 0.0001.

To define the molecular mechanism(s) through which carmofur regulated the cell cycle in TMZ-resistant GBM cells, we sought to identify genes that were decreased by carmofur treatment and upregulated in TMZ-resistant cells in comparison to parental, TMZ-sensitive cells. To identify differences between the parental and TMZ-resistant GBM cells, we used our previously published RNA-sequencing data to identify genes which were upregulated in the U251T (TMZ-resistant) cells compared to the U251P (TMZ-sensitive) cells basally (Fig. S4, Table S1). Then, we identified genes which were both upregulated in the U251T cells and were decreased in the U251T cells treated with carmofur for 48 hours (Fig. 4A, B). Of these 25 genes, 6 were cell cycle-related (*SMC2, CKAP2L, CDKN2C, ERCC6L, E2F8, KIF4B*) (Fig. 4C). Of those 6 genes, we chose to assess mRNA expression of E2F8 because it has been previously associated with radiation resistance in GBM [23]. We confirmed E2F8 mRNA and protein were elevated in the U251T cells (Fig. 4D, E, S5A, B). While there was no statistically different change in the levels of E2F8 mRNA in U251P cells treated with carmofur (Fig. 4F, G), E2F8 was significantly decreased in carmofur-treated U251T cells (Fig. 4H, I).

**Fig. 5:**
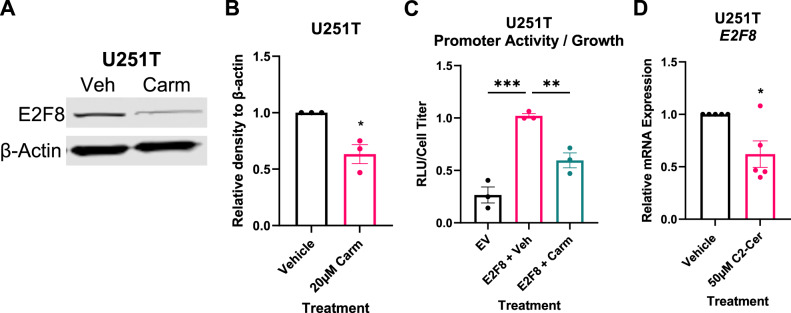
C2-ceramide or carmofur treatment decrease E2F8 expression or promoter activity, respectively. **A** E2F8 protein expression in U251T cells treated with 20 µM carmofur. **B** Quantification of E2F8 immunoblot normalized to β-actin (*n* = 3). **C** E2F8 promoter activity in U251T normalized to growth following 24 hours treatment with vehicle or 20 µM carmofur (*n* = 3 independent experiments). Comparisons were made to both empty vector (EV) and vehicle control. **D** *E2F8* mRNA expression in U251T following 48 hours treatment with 50 µM C2-ceramide (*n* = 5 independent experiments). Data were analyzed by independent *t* test or one-way ANOVA followed by Dunnett’s multiple comparisons test. Data are shown as mean ± SEM. * for *p* < 0.05, ** for *p* < 0.01, *** for *p* < 0.001.

Similarly, E2F8 protein expression was significantly decreased in carmofur-treated U251T cells (Fig. 5A, B, S6), while E2F8 expression was unchanged in carmofur-treated U251P cells (Fig. S5A, C). Next, to determine whether carmofur causes changes in E2F8 transcription or mRNA degradation, we utilized a LightSwitch reporter assay for E2F8. When carmofur was added, activity of the E2F8 promoter decreased significantly (Fig. 5C). Finally, to confirm the mechanism by which E2F8 transcription is altered, C2-ceramide was added directly to U251P and U251T cells based on previously published concentrations which induced cell death in GBM cell lines at 72 h [24]. U251P cells showed no change in E2F8 mRNA expression with the C2-ceramide treatment (Fig. S5E) while the U251T cells had significantly decreased E2F8 mRNA expression (Fig. 5D). Protein expression for U251T treated with C2-ceramide showed trending decreases but significance was limited by variability in biological replicates although there were no statistical outliers (Fig. S5A, D). Together, these data suggest that carmofur decreases E2F8 transcription to reduce E2F8 expression and shift the cell cycle in TMZ-resistant GBM cells, but further mechanistic studies are needed.

### STRING analysis indicates E2F8 is a possible hub for carmofur-affected genes in TMZ-resistant GBM cells

**Fig. 6:**
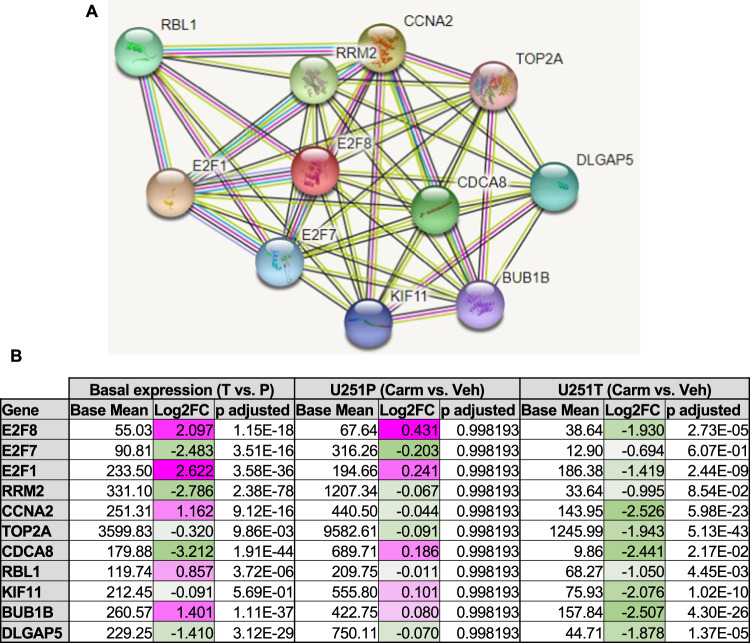
E2F8 as a hub for Carmofur affected genes. **A** String analysis for E2F8 (https://string-db.org/) (accessed on 8 August 2022). **B** Base mean, Log2FC, and *p* adjusted value for each protein identified to interact with E2F8.

Our data suggest that E2F8 elevation could be a mechanism by which U251T cells are resistant to TMZ but vulnerable to ASAH1 inhibition. To further investigate this possibility, we used STRING analysis to look at other proteins which interact with E2F8 (Fig. 6A). We compiled the base mean, log_2_ fold change, and *p* adjusted for each of the genes identified from the STRING analysis (Fig. 6B). Four of the E2F8 interacting genes (*E2F1, CCNA2, RBL1, BUB1B*) were also upregulated in the U251T cells compared to U251P. Interestingly, 9 of the 12 E2F8 interacting genes (*E2F1, RRM2, CCNA2, TOP2A, CDCA8, RBL1, KIF11, BUB1B, DLGAP5*) were significantly decreased in the carmofur-treated U251T cells. Together, these data suggest that the downregulation of E2F8 and related signaling pathways by carmofur could be an important mechanism through which TMZ-resistant GBM cell growth is impacted by carmofur. Furthermore, these data suggest a possible mechanism where the interaction between ASAH1 and E2F8 is specific to TMZ-resistant GBM cells.

## Discussion

Our work has identified ASAH1 as a novel target for recurrent GBM based on patient mRNA expression and in vitro analysis. Based on the data presented here, treatment of TMZ-resistant cells with carmofur leads to cell cycle changes and increased cell death. Previously, we showed that carmofur decreased the migration of primary GBM cells [8]. When analyzed by RNA-sequencing, we found unique pathways that were altered by carmofur in the two cell types. Interestingly, we identified E2F8 as a transcription factor which is upregulated in the TMZ-resistant cells but is decreased by carmofur treatment. These findings suggest that carmofur and the increase in ceramides have a differential effect on the parental GBM cells as compared to the TMZ-resistant GBM cells, but future lipidomic analysis is still needed to confirm this effect.

The E2F transcription factors control expression of genes required for cell cycle progression and are downstream of the retinoblastoma (RB) 1 gene. E2F8, specifically, is targeted by other E2F family members and E2F8 expression increases throughout the cell cycle [25]. While previous work in fibroblasts suggested that forced expression of E2F8 reduced cell proliferation through a negative feedback loop [25], additional research in esophageal squamous cell carcinoma identified E2F8 as a promoter of proliferation through CCND1/p21 signaling [26]. Similar conclusions were drawn from studies in papillary thyroid cancer [27]. In GBM, E2F8 expression is associated with decreased median survival, and overexpression of E2F8 increased cell growth [23]. Of importance to our findings, the same study found that E2F8 knockdown sensitized GBM cells to radiation [23]. One of the important points not explored by our work is, what is the triggering event; change in E2F8 expression or cell cycle? Since E2F8 is intricately linked to the cell cycle, there is a possibility that carmofur alters the cell cycle through another mechanism, but E2F8 decreases because less cells are progressing to the S-phase. Induction of ceramides can also lead to the dephosphorylation of RB causing cell cycle arrest through elevated expression of p21 [28].

Another key contributor to differential responses of GBM cells may be p53 status. According to TCGA data, p53 is mutated or deleted in 28% of GBM patients [29]. In colon cancer, ASAH1 expression negatively correlated with p53 activity [3]. Consistently, ceramides have been shown to induce p53 expression to trigger apoptosis [30]. We acknowledge that the differential responses seen in GBM PDX cells could be attributed to an interaction between p53 and ASAH1, though it has been reported that ceramides can also induce cell death in glioma cells through p53-independent mechanisms [31]. Future studies will address this discordance in the field literature.

Here and previously, we have shown sensitivity to carmofur in U251T (p53 mutant), Jx22T (p53 wildtype), and D456 (p53 deleted) [8]. While the IC50s vary among the cell types, all remain sensitive to carmofur between 5 and 20 µM. Moreover, trending decreases in pathways associated with DNA ionizing radiation (IR)-damage and cellular response via ATR as well as other pathways associated with DNA repair. Some of the significantly decreased genes included those associated with homologous recombination (BRCA1, RAD51), base excision repair (PARP1, PARP2), and mismatch repair (MSH6). Potential effects on the DNA damage response could be linked to the expression of E2F8. Both E2F7 and E2F8 are elevated following treatment with DNA damaging agents, and they repress E2F1-dependent apoptosis [32]. Due to these connections, future work will consider whether inhibition of ASAH1 can sensitize GBM cells to TMZ through inhibition of the DNA damage response. Additionally, further strengthening of the mechanism proposed here will include knockdown or overexpression of E2F8 to assess changes in sphingolipid metabolism in TMZ-resistance cells. We may also explore other overlapping genes identified in Fig. 4.

Although ASAH1 expression did not increase in our immunoblot analyses, ASAH1 expression was consistent between parental and TMZ-resistant cells suggesting that ASAH1 is still an appropriate target in recurrent GBM patients. One explanation for the differences between our in silico and in vitro analyses is the presence of ASAH1 in the tumor microenvironment. Previous reports suggest that secretion of ASAH1 into culture media occurs following radio therapy [9]. Based on this premise, we interrogated the expression of *ASAH1* in recurrent GBM patients using publicly available single-cell RNA-sequencing data through the Brain Immune Atlas [33]. ASAH1 was highly expressed in the macrophage and monocyte populations of recurrent GBM patients compared to other immune populations (Fig. S7). Therefore, inhibiting ASAH1 pharmacologically may have additional benefits within the tumor microenvironment such as decreasing macrophage recruitment [34]. Future studies using carmofur in immune-competent intracranial models are needed to determine if carmofur improves the survival of GBM-bearing mice.

Overall, our results suggest that targeting the altered sphingolipid balance in recurrent GBM may be an effective strategy to reduce growth specifically by targeting E2F8 expression in the TMZ-resistance cells.

## Methods

### Analysis of publicly available patient mRNA

Patient mRNA data were accessed from the Chinese Glioma Genome Atlas (CGGA) [16] and The Cancer Genome Atlas (TCGA) using the Gliovis portal [35] (http://gliovis.bioinfo.cnio.es). mRNA expression for primary and recurrent glioma patients for each ceramidase was assessed using CGGA data. Similar analyses were conducted in TCGA comparing MGMT promoter-methylated and -unmethylated glioma patient mRNA samples. Comparisons for each were plotted and analyzed using independent t-test in GraphPad v9 (Graphpad Software, San Diego, CA, USA). Single-cell data and plots were obtained from Brain Immune Atlas [33] (https://www.brainimmuneatlas.org/).

### Cell culture

U251P (TMZ-sensitive) and U251T (TMZ-resistant) cell lines were obtained from Dr. Corinne Griguer at the University of Iowa (previously at UAB). JX22P and JX22T GBM cells were derived from patient-derived xenografts (PDXs) that were from Dr. Jann Sakaria at the Mayo Clinic. PDXs were propagated in Balbc nu/nu mice and maintained as described previously [8] in accordance with approval from the University of Alabama at Birmingham Institutional Animal Care and Use Committee. All cell lines and PDX-derived cells were authenticated and cultured in serum-free media (Brain Tumor Initiating Cell or BTIC media) that was collected for mycoplasma testing. BTIC media included DMEM/F12 supplemented with Gem 21 Neuroplex without vitamin A (Gemini Bioproducts, West Sacramento, CA, USA, cat# 400-161), 10 ng/mL of epidermal growth factor and fibroblast growth factor (Gemini Bioproducts, West Sacramento, CA, USA, cat# 300-110 P and 300-112 P), 1% sodium pyruvate (Gibco, Waltham, MA, USA, cat# 11360070), and 100 U/mL penicillin and 100 µg/mL streptomycin (Gibco, Waltham, MA, USA, cat# 15-140-122).

### Cell growth assessment

For growth assays, cells were plated at 2000 cells/well in a 96-well plate. Following recovery, cells were treated with vehicle (DMSO) or the indicated concentrations of carmofur (Selleckchem, Houston, TX, USA, cat# S1289) dissolved in DMSO. After 7 days, images were obtained using EVOS XL Core (Life Technologies, Carlsbad, CA, USA) to show changes in cell morphology and confluence visually followed by analysis for cell viability using Cell Titer Glo (Promega, Madison, WI, USA) as previously described [8].

### Cell cycle analysis

To assess cell cycle changes, cells were plated at 1 million cells per 100 mm plate. The following day, cells were treated with vehicle (DMSO) or their respective IC50s for carmofur as indicated in the figure legend. After 48 h, cells were collected and separated using Accutase (Gibco, Waltham, MA, USA, cat# A11105-01). Then, cells were fixed with 70% ethanol containing 10% FBS while vortexing to prevent clumping. Cells were stored at 4 °C until ready for analysis. After collection of three biological replicates, cells were washed and stained with propidium iodide (PI) at 50 µg/mL (Abcam, Cambridge, UK, cat #ab14083) in the presence of PureLink RNase A at 1 mg/mL (Invitrogen, Waltham, MA, USA cat# 12091-021) for one hour before analyzing using flow cytometry. FACSymphony A5 Cell Analyzer (BD Biosciences, San Jose, CA, USA) was used to assess DNA quantity with the PI staining. Cell cycle phases were determined based on peaks representing single stranded and double stranded DNA in FlowJo v10.8.1. Percentages of cells in each phase of the cell cycle for each treatment were plotted together for the three biological replicates. Independent t-tests were used to determine if there was a significant difference between the carmofur and vehicle treatments.

### Apoptosis analysis

To assess apoptosis, cells were plated at 5000 cells/well, allowed to recover, and treated with carmofur at IC25, IC50, and IC75. After 48 h in the presence of carmofur or vehicle, apoptosis was assessed using the Annexin V/Necrosis Assay (Promega, Madison, WI, USA cat# PRJA1011) multiplexed with Cell Titer Glo to normalize for cell number. Data were analyzed by one-way ANOVA followed by Dunnett’s multiple comparisons test.

### RNA-sequencing

RNA-sequencing of U251T cells treated with vehicle or 20 µM carmofur for a total of 48 h was conducted as in our previous publication with parental U251 cells [8]. Matching replicates for both U251P and U251T were collected at the same time. Webgestalt (http://www.webgestalt.org) (Accessed on 20 October 2021) [22] was used to analyze differentially expressed genes (base mean >10, *p* value (unadjusted) <0.05, log_2_ fold change >1.5 or <-1.5). Gene set enrichment analysis (GSEA) was conducted using the Wikipathway Cancer functional database with a minimum of 5 genes/pathway. A second analysis of differentially expressed genes was conducted using Qiagen Ingenuity Pathway Analysis (https://digitalinsights.qiagen.com/IPA) (accessed on 21 February 2022) [36]. For the comparison of U251T to U251P cells, the same criteria for Webgestalt input were used with the exception of a *p* adjusted value of <0.05 and 10 genes/pathway as there was a higher number of differentially expressed genes. Data is available through Gene Expression Omnibus accession number GSE179087.

### qPCR analysis

To validate RNA-sequencing results, separate but identically treated samples were collected using the Qiagen RNA isolation kit (Germantown, MD, USA, cat# 74106). Additional samples were collected from cells treated with C2-ceramide (D18:1/2:0) (Cayman Chemical, Ann Arbor, MI, USA, cat# 62510) at the indicated concentrations for 48 hours. iScript cDNA synthesis reaction (BioRad, Hercules, CA, USA, cat# 170-8891) was used to generate cDNA before conducting RT-qPCR using SsoAdvanced Universal SYBR Green Supermix (BioRad, Hercules, CA, USA, cat# 172-5274). β-*ACTIN* (FWD: AGA AAA TCT GGC ACC ACA CC; REV: AGA GGC GTA CAG GGA TAG CA) and *E2F8* (FWD: CCT GAG ATC CGC AAC AGA GAT; REV: AGA TGT CAT TAT TCA CAG CAG GG) primers were purchased from IDT (Coralville, IA, USA). Delta CT values normalized to β-*ACTIN* were used to assess relative changes in gene expression and were subject to statistical analysis.

### Immunoblot analysis

Lysate preparation and immunoblot analysis were conducted, as previously described [8]. Briefly, U251T cells were serum-starved overnight in the presence of 20 µM carmofur to synchronize the cell cycle followed by a second carmofur treatment in DMEM/F12 with 10% FBS (Peak Serum, Wellington, CO, USA, cat# PS-FB2) for a total of 48 hours with carmofur. A total of 40 µg of protein were loaded for immunoblot analysis. E2F8 and ASAH1 polyclonal antibodies were purchased from Proteintech (Rosemont, IL, USA, cat# 13425) and Invitrogen (Waltham, MA, USA, cat# PA5-20574), respectively.

### E2F8 promoter activity analysis

Cells were plated at 10000 cells/well of a 96-well plate on Geltrex (Gibco, Waltham, MA, USA cat# A1413302). The following day, cells were transfected with either LightSwitch Promoter Empty Vector (SwitchGear Genomics, Menlo Park, CA, USA cat# S790005) or E2F8 Promoter Reporter (SwitchGear Genomics, Menlo Park, CA, USA cat# 32001) using U251 Cell Avalanche Transfection Reagent (EZ Biosystems, College Park, MD, USA, cat# EZT-U251-1). 40 ng of DNA was added to each well with reagent at a 1:5 ratio of DNA to reagent in BTIC media without penicillin/streptomycin. After 6 h in the presence of the transfection reagent, the media was changed to complete BTIC media. The following day, E2F8 promoter-expressing cells were treated with vehicle or 20 µM carmofur. After 24 h after the carmofur treatment, the LightSwitch Luciferase Assay (SwitchGear Genomics, Menlo Park, CA, USA cat# LS010) was conducted as indicated by the manufacturer. Wells with matching treatments were assayed with Cell Titer Glo to control for changes in cell growth caused by the transfection.

### Statistical analysis

Biological triplicates were conducted for all experiments with 3 technical replicates for each individual experiment except in qPCR analyses where 2 technical replicates were used for each biological replicate. Prism v9 was used for analyses with relevant statistical tests completed as indicated in each figure legend.

### Supplementary information

Supplemental Material

All Figures Together as PDF

## Data Availability

RNA-sequencing data are available through Gene Expression Omnibus accession number GSE179087.
