# Supplementary material for: Carmofur prevents cell cycle progression by reducing E2F8 transcription in temozolomide-resistant glioblastoma cells
Source: Cell Death Discov. 2023 Dec 12;9:451. doi: 10.1038/s41420-023-01738-x (PMC10716181; doi:10.1038/s41420-023-01738-x)
Supplement: Supplementary file 1 — Supplemental Material [file 41420_2023_1738_MOESM1_ESM.pdf]

Gene Set	Description	Size	Leading Edge Number	ES	NES	P Value	FDR
WP3859	TGF-B Signaling in Thyroid Cells for Epithelial-Mesenchymal Transition	10	10	0.41255	1.2649	0.2025	1
WP4206	Hereditary leiomyomatosis and renal cell carcinoma pathway	10	9	0.33391	1.0345	0.3641	1
WP4249	Hedgehog Signaling Pathway	15	2	0.29043	1.0313	0.42416	1
WP3617	Photodynamic therapy-induced NF-kB survival signaling	12	4	0.23882	0.7785	0.74877	1
WP45	G1 to S cell cycle control	29	2	0.1744	0.7561	0.79753	1
WP3972	PDGFR-beta pathway	12	6	0.23357	0.7526	0.77017	1
WP1539	Angiogenesis	12	7	0.18061	0.5879	0.9525	1
WP61	Notch Signaling Pathway Netpath	19	16	0.13555	0.5117	0.97927	1
WP179	Cell Cycle	44	2	0.09449	0.4382	1	0.99752
WP3844	PI3K-AKT-mTOR signaling pathway and therapeutic opportunities	16	9	-0.3742	-1.259	0.20202	0.59865
WP707	DNA Damage Response	28	6	-0.3268	-1.272	0.16272	0.60929
WP619	Type II interferon signaling (IFNG)	11	7	-0.4343	-1.278	0.16275	0.64054
WP585	Interferon type I signaling pathways	21	10	-0.3633	-1.298	0.16172	0.636
WP4205	MET in type 1 papillary renal cell carcinoma	23	7	-0.3851	-1.425	0.093802	0.53673
WP241	One Carbon Metabolism	12	6	-0.4681	-1.431	0.098684	0.60574
WP4263	Pancreatic adenocarcinoma pathway	37	12	-0.3826	-1.621	0.01248	0.26324
WP4255	Non-small cell lung cancer	35	13	-0.3954	-1.634	0.012559	0.32067
WP1601	Fluoropyrimidine Activity	18	13	-0.5249	-1.846	0.003373	0.07029
WP254	Apoptosis	30	16	-0.5073	-2.028	0	0.01378

Supplemental Table 1. U251T cells have decreased expression of genes related to apoptosis compared to U251P. Table shows differentially expressed pathways as well as their enrichment score (ES), normalized enrichment score (NES), p value, and false discovery rate (FDR).

A

Gene	Protein	Localization
ASAH1	Acid Ceramidase	Lysosome
ASAH2	Neutral Ceramidase	Plasma membrane
ACER1	Alkaline Ceramidase 1	Endoplasmic reticulum
ACER2	Alkaline Ceramidase 2	Golgi complex
ACER3	Alkaline Ceramidase 3	Endoplasmic reticulum, Golgi complex

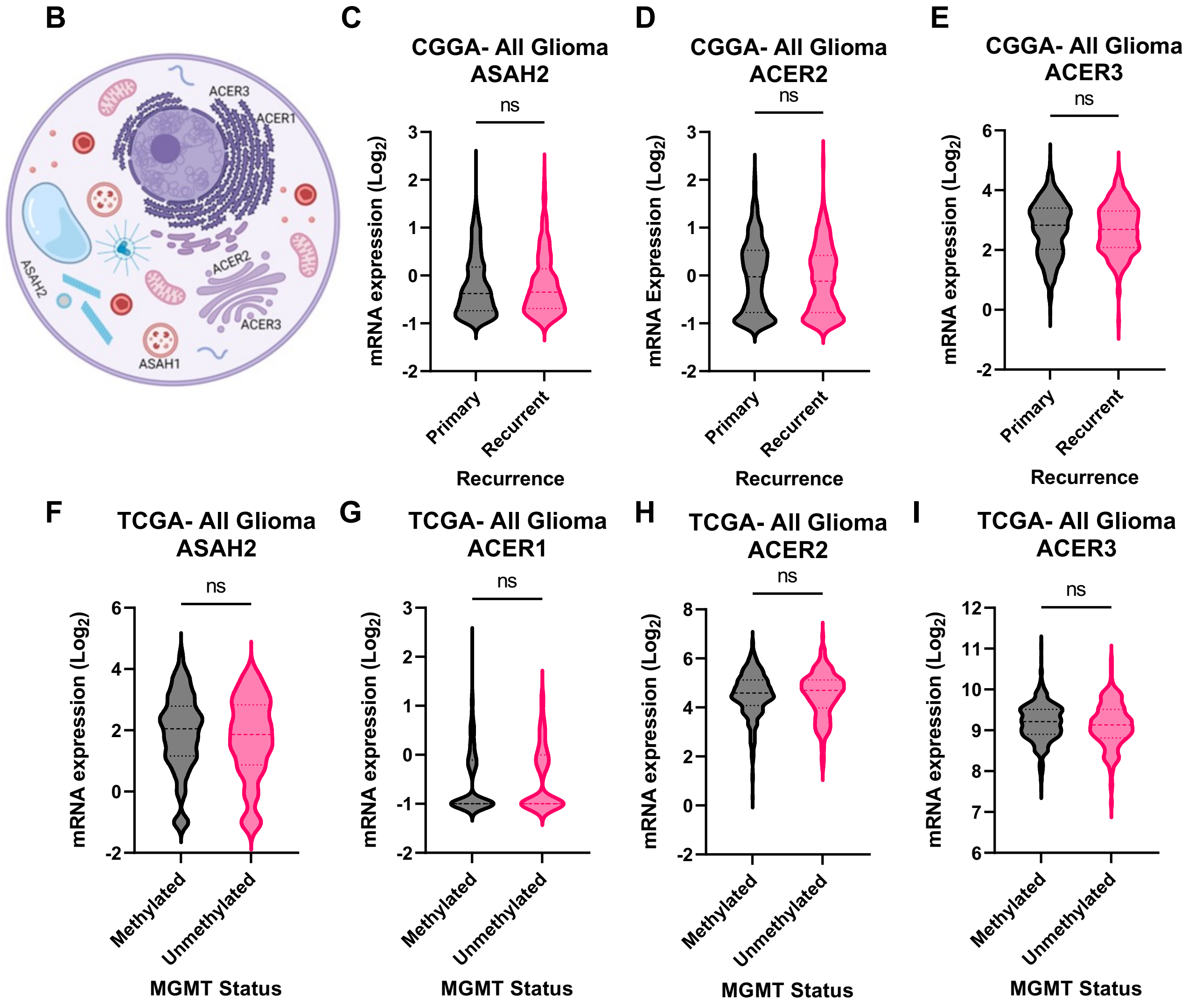

Supplemental Figure 1. Expression for ceramidases in recurrent and MGMT unmethylated gliomas. (A) Table and (B) schematic showing gene names and localization for ceramidases. mRNA expression for ceramidases in primary and recurrent glioma patients was accessed using GlioVis (http://gliovis.bioinfo.cnio.es) (Accessed on 28 December 2021). Comparisons were made between recurrent and primary glioma patients for (C) *ASAH2*, (D) *ACER2*, and (E) *ACER3* (n=651 for primary, n=333 for recurrent). mRNA expression for ceramidases in MGMT promoter-unmethylated glioma. Comparison of gene expression for methylated and unmethylated gliomas in TCGA dataset for (F) *ASAH2*, (G) *ACER1*, (H) *ACER2*, (I) *ACER3* (n=477 for methylated, n=161 for unmethylated). Data were analyzed using an independent t-test for C-H, and data were analyzed using non-parametric, Mann-Whitney t-test for I. Data are shown as mean ± SEM.

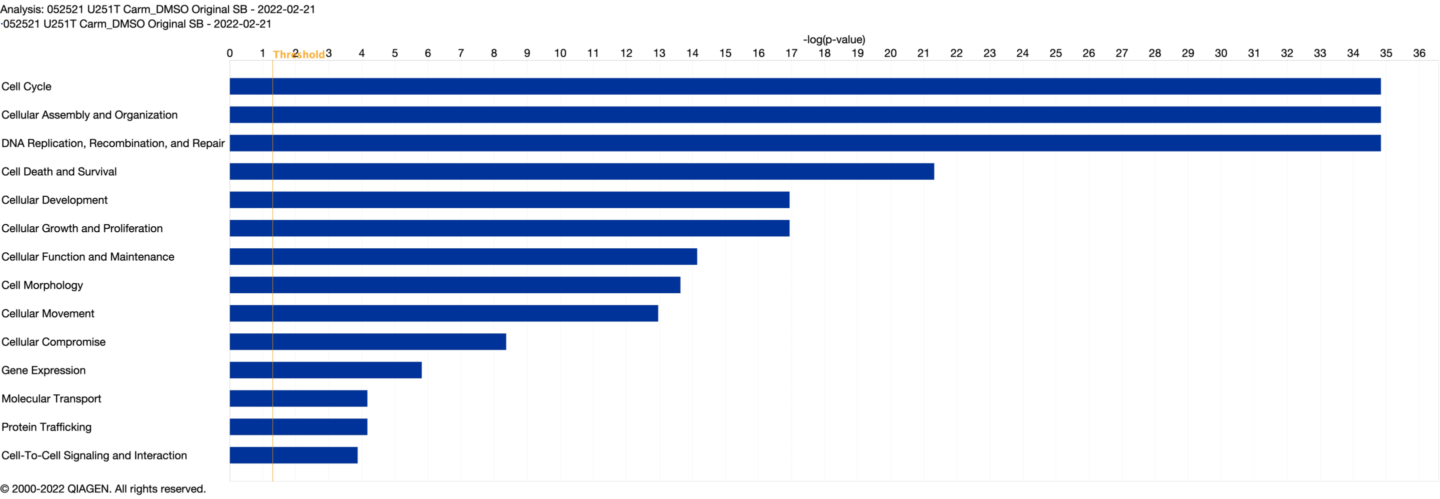

Supplemental Figure 2. Biological pathways affected by carmofur treatment from Ingenuity Pathway Analysis. Biological pathways and their z-scores for RNA-sequencing analysis of U251T cells treated with carmofur (https://digitalinsights.qiagen.com/IPA) (accessed on 21 February 2022).

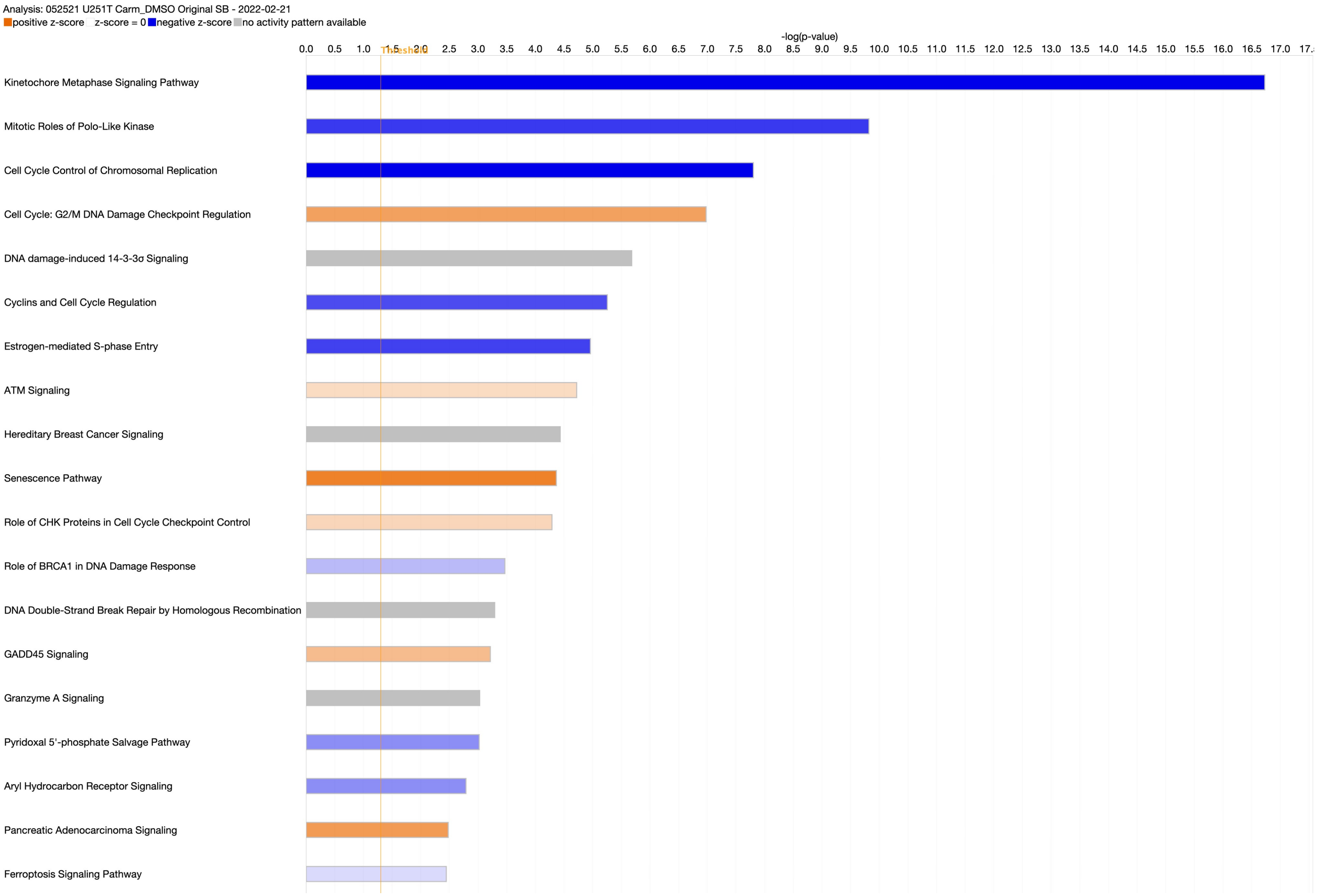

Supplemental Figure 3. Ingenuity pathway analysis shows decrease in cell cycle pathways with carmofur treatment. Canonical pathways and their z-scores for RNA-sequencing analysis of U251T cells treated with carmofur (https://digitalinsights.qiagen.com/IPA) (accessed on 21 February 2022).

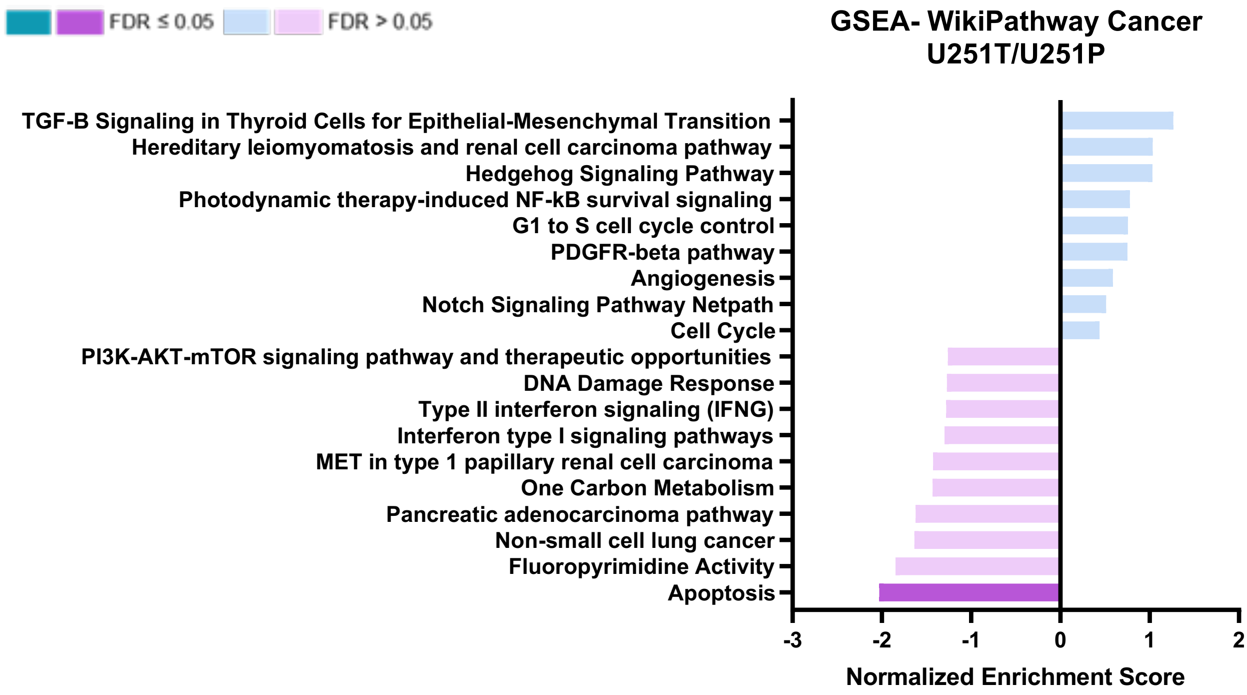

Supplemental Figure 4. U251T cells have decreased expression of apoptosis-related genes. RNA-sequencing data was analyzed to assess differences basally between U251T vehicle and U251P vehicle (n=3). Gene set enrichment analysis (GSEA) was conducted for genes with a log2 FC >1.5 or <-1.5 using Webgestalt (http://www.webgestalt.org) (Accessed on 14 June 2022). Wikipathway cancer functional database and a minimum of 10 genes/pathway were used for the analysis. Selection criteria for input genes included base mean >10 and a p adjusted value <0.05.

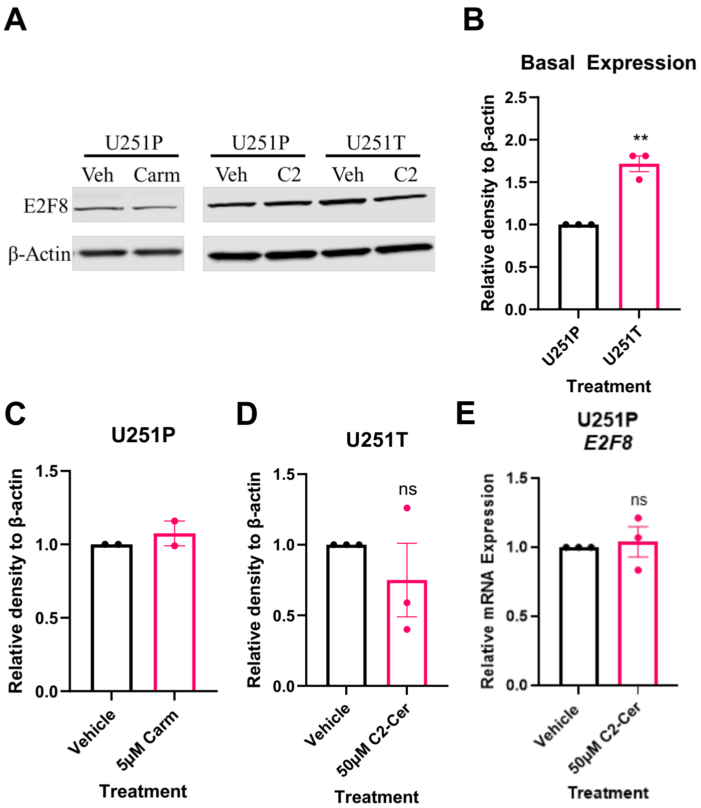

Supplemental Figure 5. E2F8 expression in U251P and T with carmofur or C2-ceramide treatment. (A) E2F8 protein expression in U251P and T treated with carmofur or C2-ceramide. (B) Quantification of E2F8 expression at baseline between U251P and T (n=3). (C) Quantification of E2F8 protein expression in U251P treated with 5 μM carmofur (n=2). (D) Quantification of immunoblot for U251T treated with 50 μM C2-ceramide (n=3). (E) U251T mRNA expression for *E2F8* following 48 hours treatment with 50 μM C2-ceramide. Densitometry was calculated in Image Studio Lite v 5.2 and normalized to β-actin. Where n=3, comparisons were made using independent t-test ± SEM. ** for p<0.01

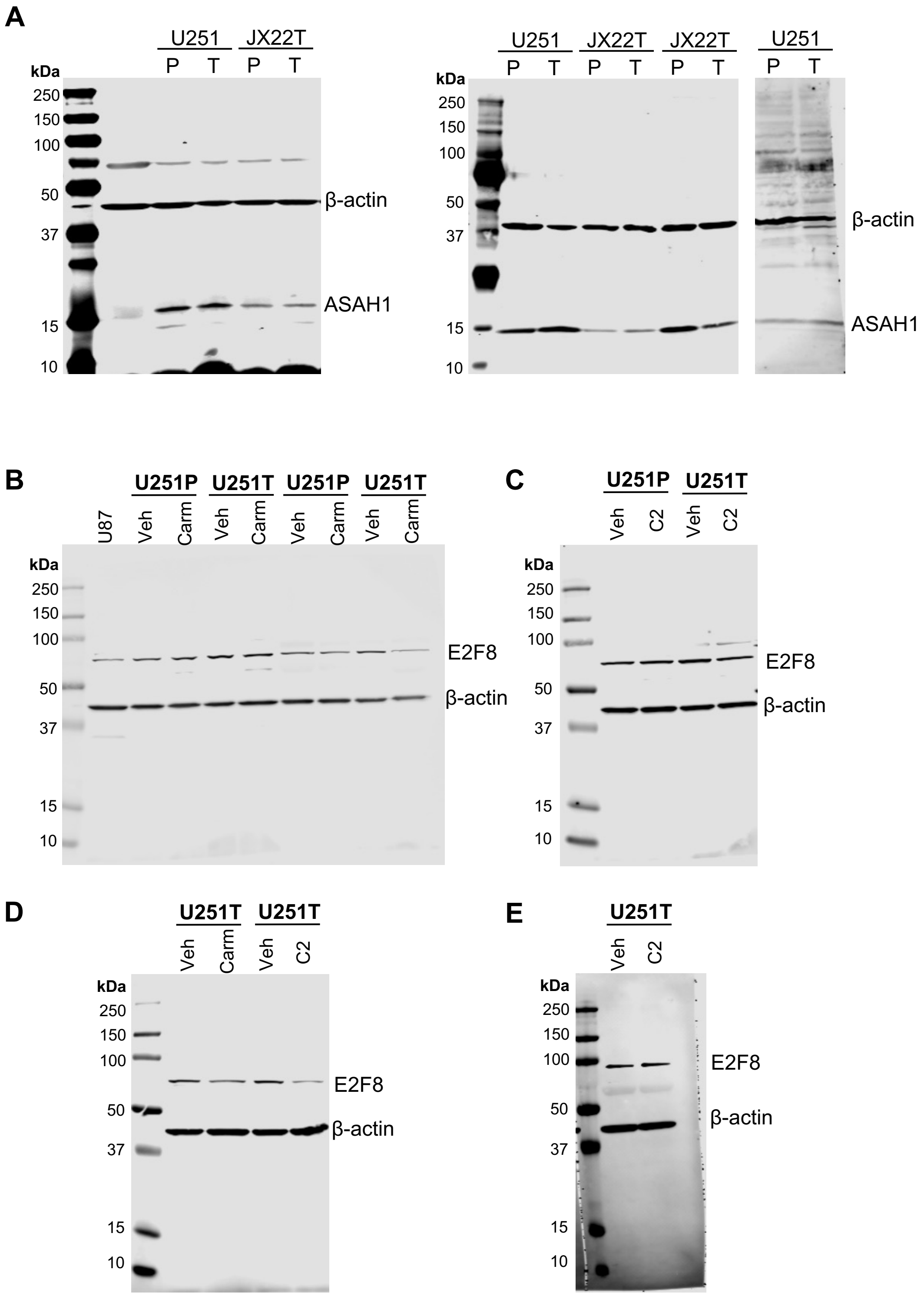

Supplemental Figure 6. Full immunoblot images. (A) Basal expression of ASAH1 in U251P and T as well as JX22P and T (n=3). (B) U251P (n=2) and T (n=3) treated with vehicle or carmofur at 5μM and 20μM for U251P and T, respectively. (C) U251P (n=1) and T (n=3) treated with vehicle or 50μM C2-ceramide. (D) Repeat of U251T cells treated with carmofur or C2-ceramide. (E) Repeat of U251T treated with C2-ceramide.

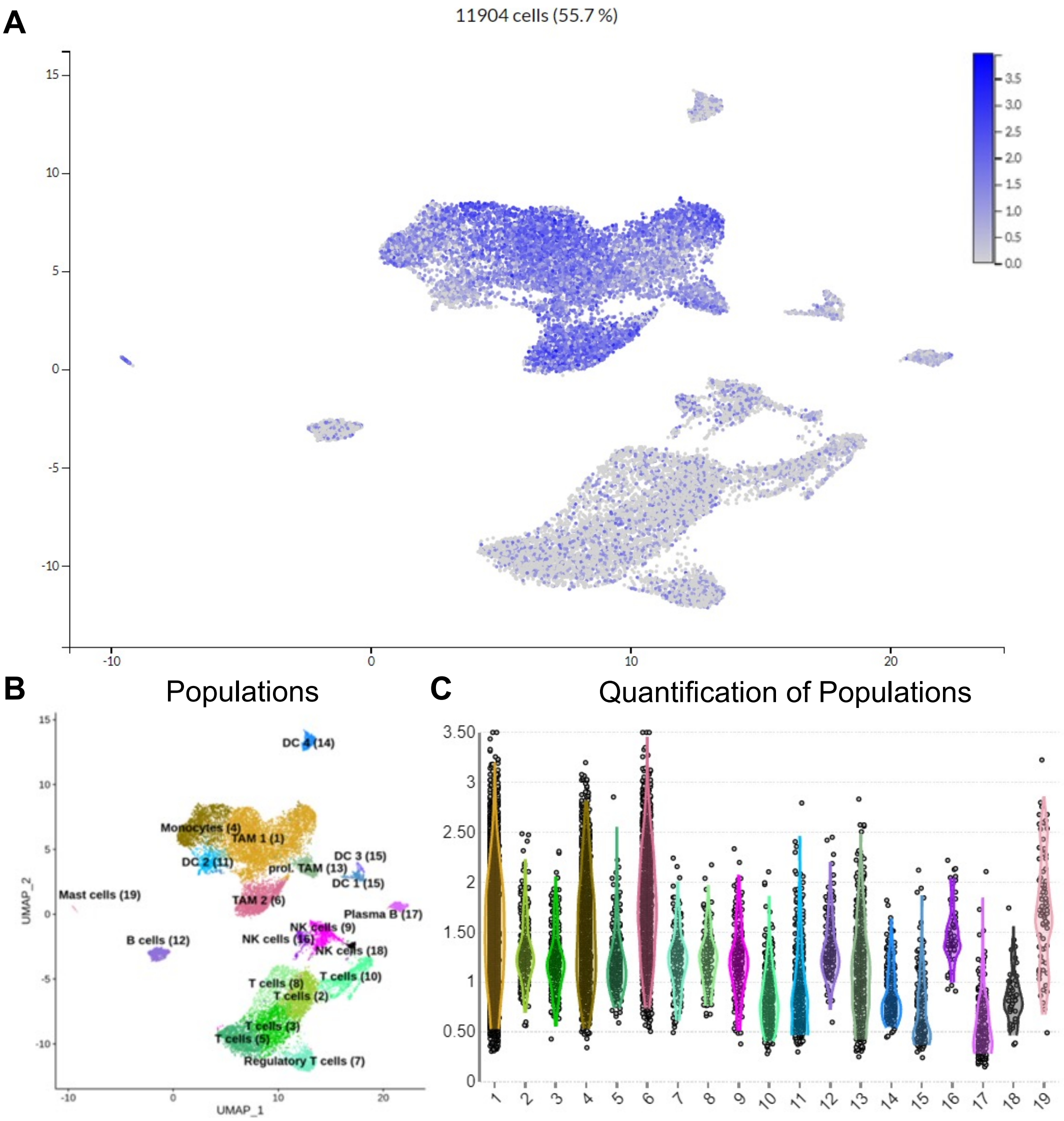

Supplemental Figure 7. *ASAH1* is highly expressed in tumor-associated macrophages (TAMs) of recurrent GBM patients. (A-B) Using publicly available single-cell RNA-sequencing data from Brain Immune Atlas (https://www.brainimmuneatlas.org/; accessed on 14 October 2021), UMAP projection for *ASAH1* shows overlap with macrophage and monocyte populations. (C) As evidenced by quantification, *ASAH1* is highly expressed in macrophages and monocytes of recurrent GBM patients.
