# Supplementary material for: Carmofur prevents cell cycle progression by reducing E2F8 transcription in temozolomide-resistant glioblastoma cells
Source: Cell Death Discov. 2023 Dec 12;9:451. doi: 10.1038/s41420-023-01738-x (PMC10716181; doi:10.1038/s41420-023-01738-x)
Supplement: Supplementary file 2 — All Figures Together as PDF [file 41420_2023_1738_MOESM2_ESM.pdf]

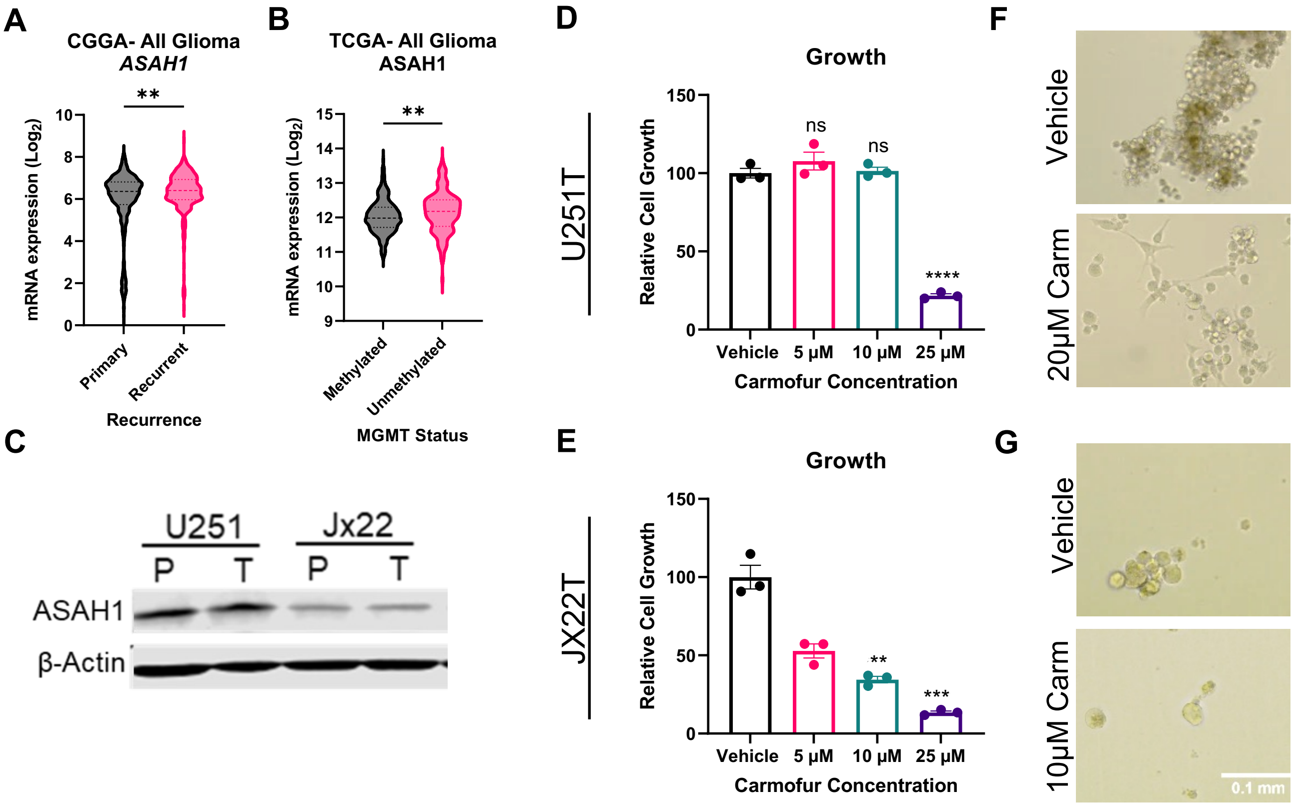
A
CGGA- All Glioma
ASAH1
**
mRNA expression (Log2)
10
8
6
4
2
0
Primary
Recurrent
Recurrence
B
TCGA- All Glioma
ASAH1
**
mRNA expression (Log2)
15
14
13
12
11
10
9
Methylated
Unmethylated
MGMT Status
C
U251
Jx22
P
T
P
T
ASAH1
β-Actin
D
U251T
Growth
Relative Cell Growth
150
100
50
0
ns
ns
****
Vehicle
5 µM
10 µM
25 µM
Carmofur Concentration
E
JX22T
Growth
Relative Cell Growth
150
100
50
0
**
***
Vehicle
5 µM
10 µM
25 µM
Carmofur Concentration
F
Vehicle
20µM Carm
G
Vehicle
10µM Carm
0.1 mm

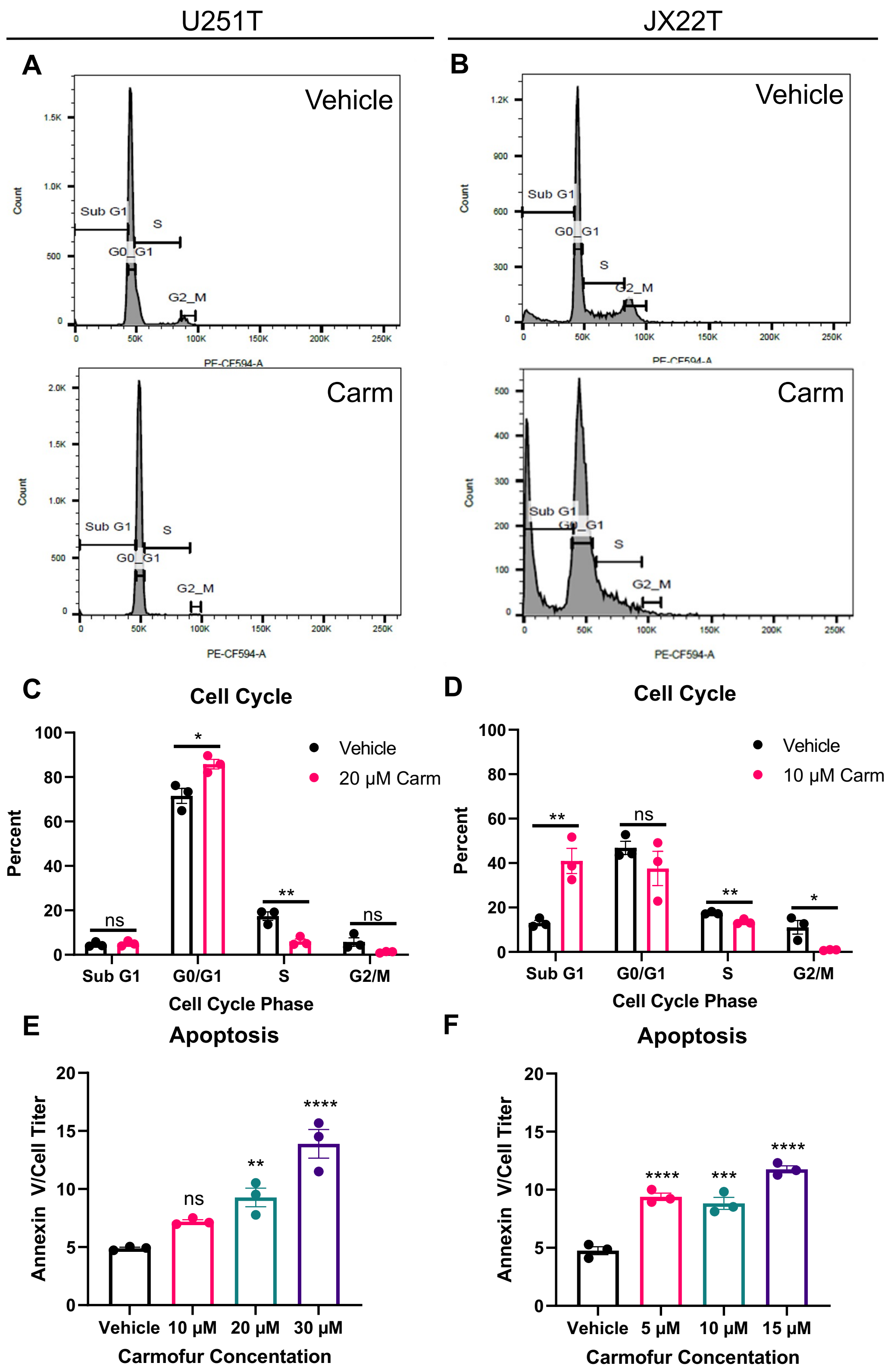
U251T
JX22T
A
B
Vehicle
Carm
Sub G1
G0_G1
S
G2_M
Count
PE-CF594-A
C
Cell Cycle
Vehicle
20 μM Carm
Percent
Sub G1
G0/G1
S
G2/M
Cell Cycle Phase
ns
*
**
D
Cell Cycle
Vehicle
10 μM Carm
E
Apoptosis
Annexin V/Cell Titer
Vehicle
10 μM
20 μM
30 μM
Carmofur Concentation
****
F
Apoptosis
5 μM
15 μM
***

A

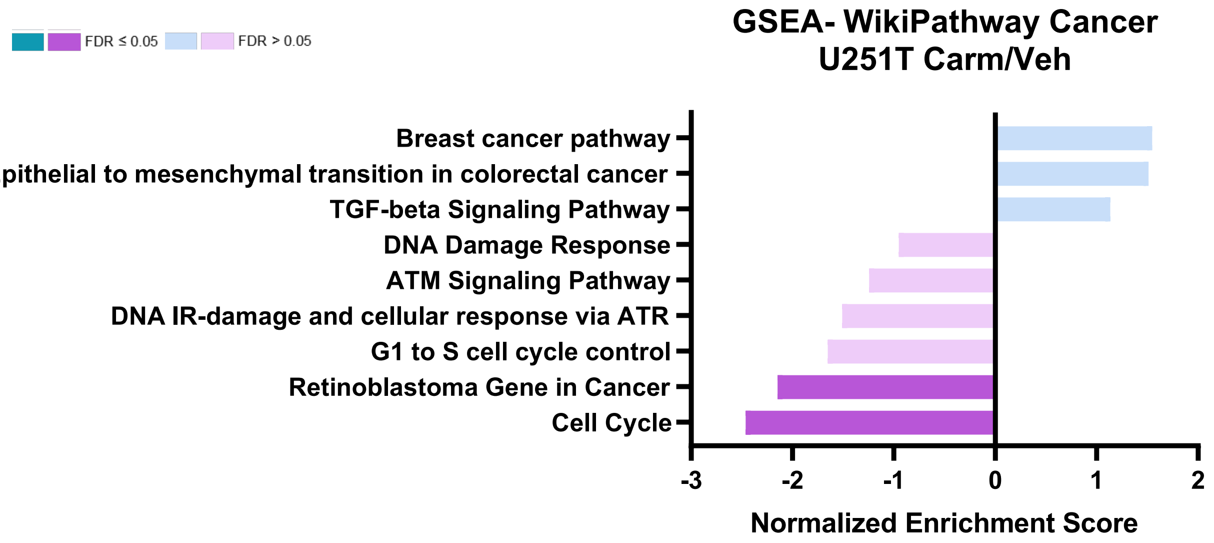

B

Gene Set	Description	Size	Leading Edge Number	ES	NES	P Value	FDR
WP4262	Breast cancer pathway	6	4	0.53301	1.548	0.04661	0.16355
WP4239	Epithelial to mesenchymal transition in colorectal cancer	5	4	0.57187	1.5119	0.06375	0.10104
WP366	TGF-beta Signaling Pathway	5	3	0.43249	1.1336	0.30081	0.30795
WP707	DNA Damage Response	10	5	-0.2714	-0.9515	0.46845	0.50208
WP2516	ATM Signaling Pathway	6	4	-0.4396	-1.2436	0.18975	0.24626
WP4016	DNA IR-damage and cellular response via ATR	7	7	-0.5043	-1.5096	0.07874	0.11149
WP45	G1 to S cell cycle control	7	5	-0.5389	-1.6529	0.0426	0.07937
WP2446	Retinoblastoma Gene in Cancer	17	12	-0.4863	-2.1478	0.00625	0.00263
WP179	Cell Cycle	14	11	-0.599	-2.4633	0	0.00132

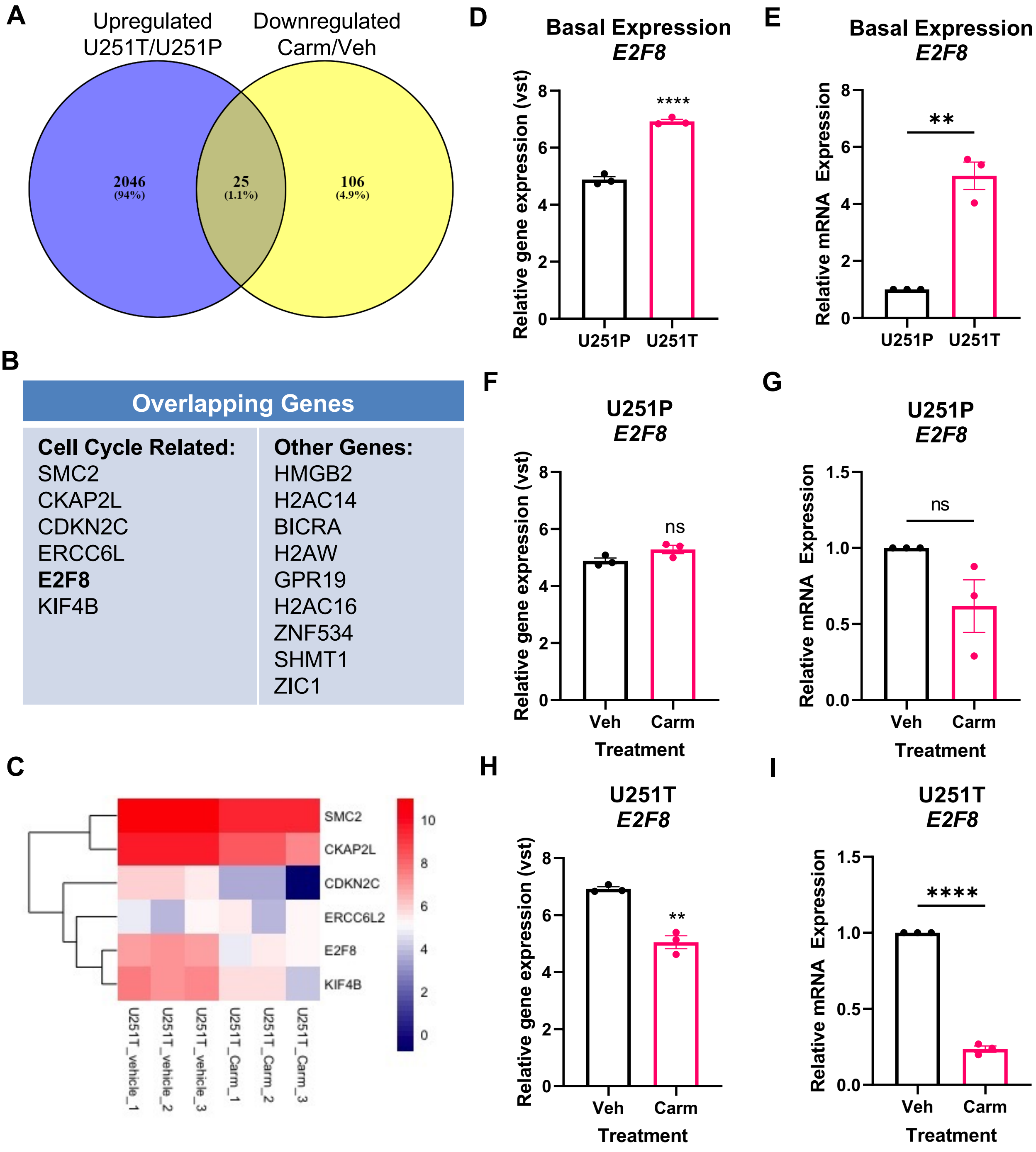
A
Upregulated
U251T/U251P
Downregulated
Carm/Veh
2046
(94%)
25
(1.1%)
106
(4.9%)
B
Overlapping Genes
Cell Cycle Related:
SMC2
CKAP2L
CDKN2C
ERCC6L
E2F8
KIF4B
Other Genes:
HMGB2
H2AC14
BICRA
H2AW
GPR19
H2AC16
ZNF534
SHMT1
ZIC1
C
SMC2
CKAP2L
CDKN2C
ERCC6L2
E2F8
KIF4B
10
8
6
4
2
0
U251T_vehicle_1
U251T_vehicle_2
U251T_vehicle_3
U251T_Carm_1
U251T_Carm_2
U251T_Carm_3
D
Basal Expression
E2F8
Relative gene expression (vst)
****
U251P
U251T
E
Basal Expression
E2F8
Relative mRNA Expression
**
U251P
U251T
F
U251P
E2F8
Relative gene expression (vst)
ns
Veh
Carm
Treatment
G
U251P
E2F8
Relative mRNA Expression
ns
Veh
Carm
Treatment
H
U251T
E2F8
Relative gene expression (vst)
**
Veh
Carm
Treatment
I
U251T
E2F8
Relative mRNA Expression
****
Veh
Carm
Treatment

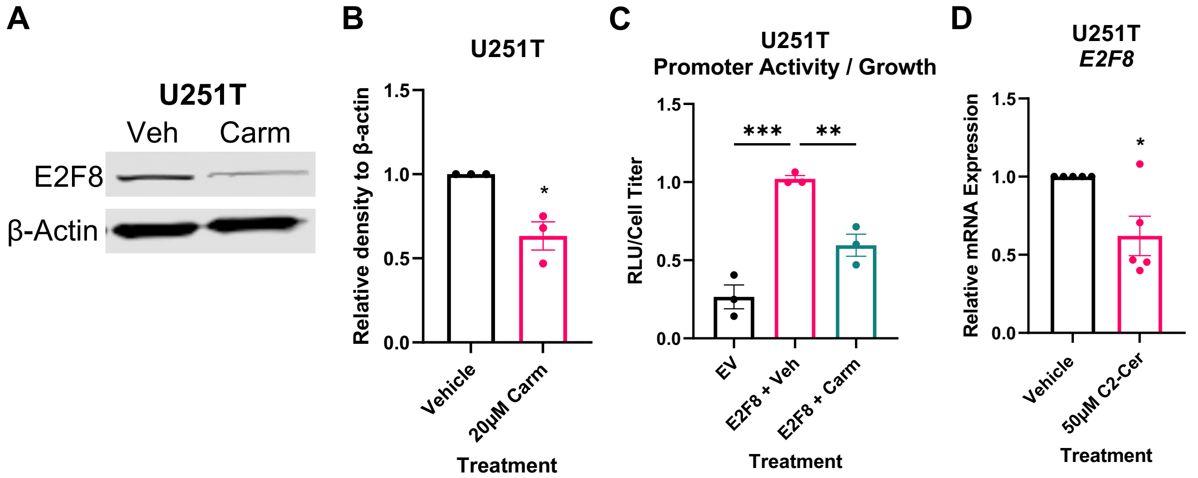
A
U251T
Veh
Carm
E2F8
β-Actin
B
U251T
Relative density to β-actin
1.5
1.0
0.5
0.0
*
Vehicle
20μM Carm
Treatment
C
U251T
Promoter Activity / Growth
RLU/Cell Titer
1.5
1.0
0.5
0.0
***
**
EV
E2F8 + Veh
E2F8 + Carm
Treatment
D
U251T
E2F8
Relative mRNA Expression
1.5
1.0
0.5
0.0
*
Vehicle
50μM C2-Cer
Treatment

A

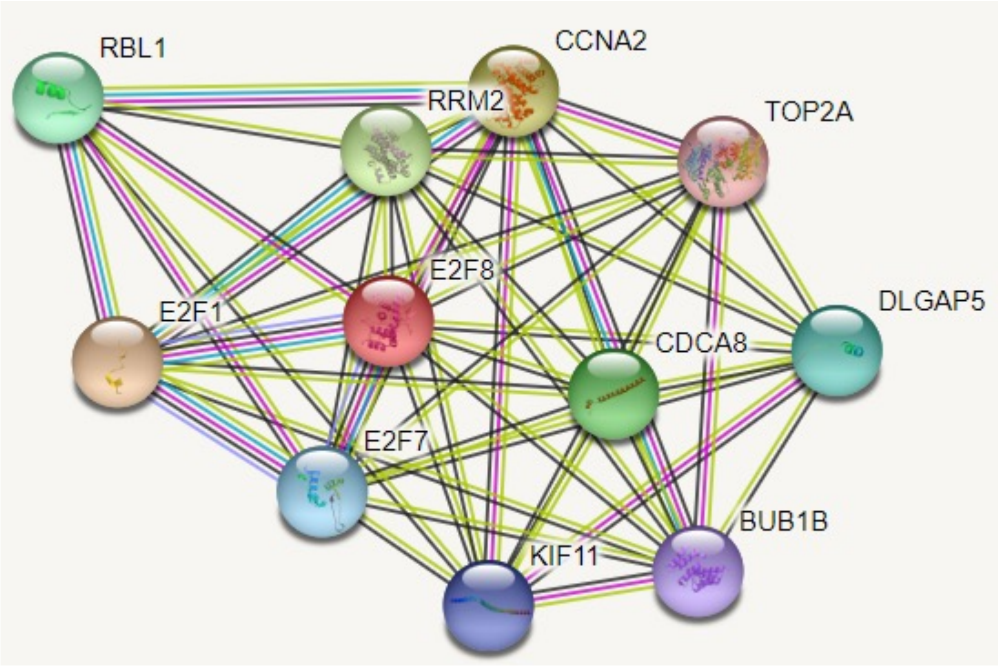

B

	Basal expression (T vs. P)			U251P (Carm vs. Veh)			U251T (Carm vs. Veh)		
**Gene**	**Base Mean**	**Log2FC**	**p adjusted**	**Base Mean**	**Log2FC**	**p adjusted**	**Base Mean**	**Log2FC**	**p adjusted**
**E2F8**	55.03	2.097	1.15E-18	67.64	0.431	0.998193	38.64	-1.930	2.73E-05
**E2F7**	90.81	-2.483	3.51E-16	316.26	-0.203	0.998193	12.90	-0.694	6.07E-01
**E2F1**	233.50	2.622	3.58E-36	194.66	0.241	0.998193	186.38	-1.419	2.44E-09
**RRM2**	331.10	-2.786	2.38E-78	1207.34	-0.067	0.998193	33.64	-0.995	8.54E-02
**CCNA2**	251.31	1.162	9.12E-16	440.50	-0.044	0.998193	143.95	-2.526	5.98E-23
**TOP2A**	3599.83	-0.320	9.86E-03	9582.61	-0.091	0.998193	1245.99	-1.943	5.13E-43
**CDCA8**	179.88	-3.212	1.91E-44	689.71	0.186	0.998193	9.86	-2.441	2.17E-02
**RBL1**	119.74	0.857	3.72E-06	209.75	-0.011	0.998193	68.27	-1.050	4.45E-03
**KIF11**	212.45	-0.091	5.69E-01	555.80	0.101	0.998193	75.93	-2.076	1.02E-10
**BUB1B**	260.57	1.401	1.11E-37	422.75	0.080	0.998193	157.84	-2.507	4.30E-26
**DLGAP5**	229.25	-1.410	3.12E-29	750.11	-0.070	0.998193	44.71	-1.878	1.37E-05

A

Gene	Protein	Localization
ASAH1	Acid Ceramidase	Lysosome
ASAH2	Neutral Ceramidase	Plasma membrane
ACER1	Alkaline Ceramidase 1	Endoplasmic reticulum
ACER2	Alkaline Ceramidase 2	Golgi complex
ACER3	Alkaline Ceramidase 3	Endoplasmic reticulum, Golgi complex

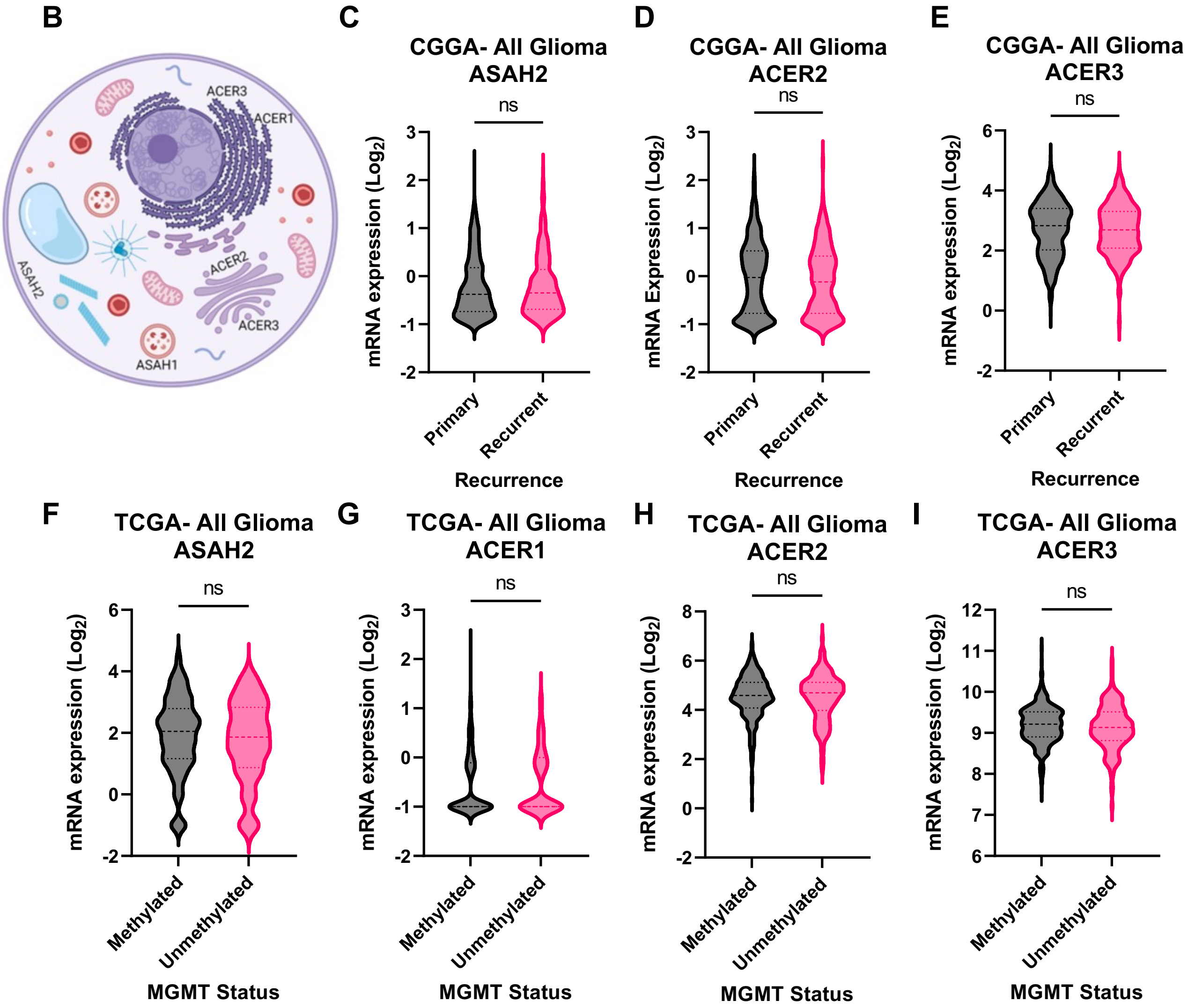

Hawkins, et al. Supplemental Figure 2

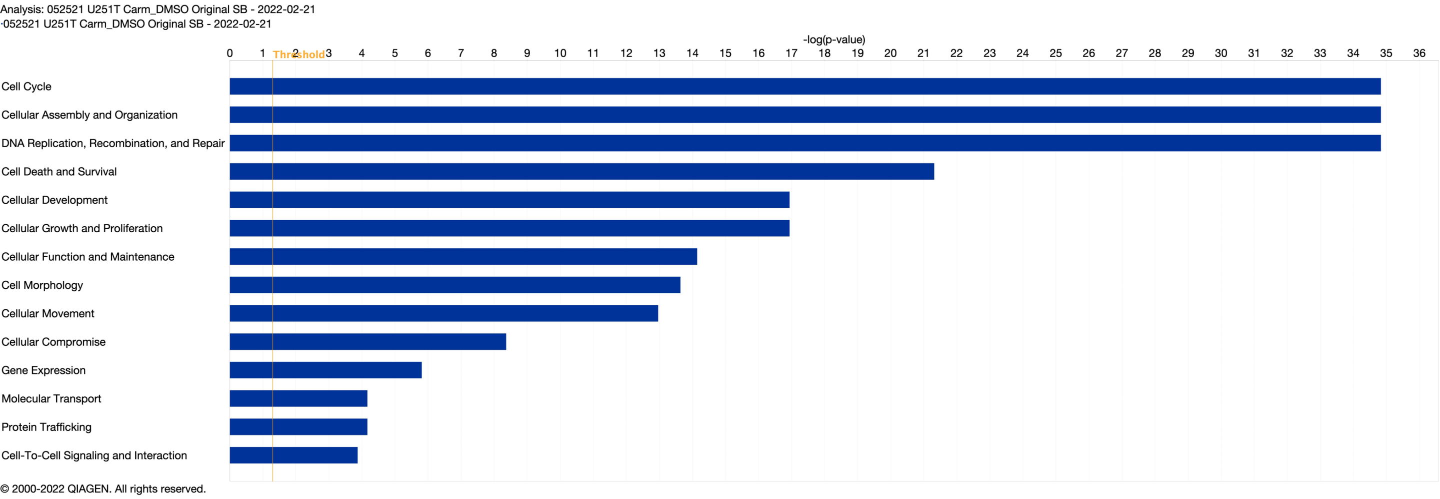

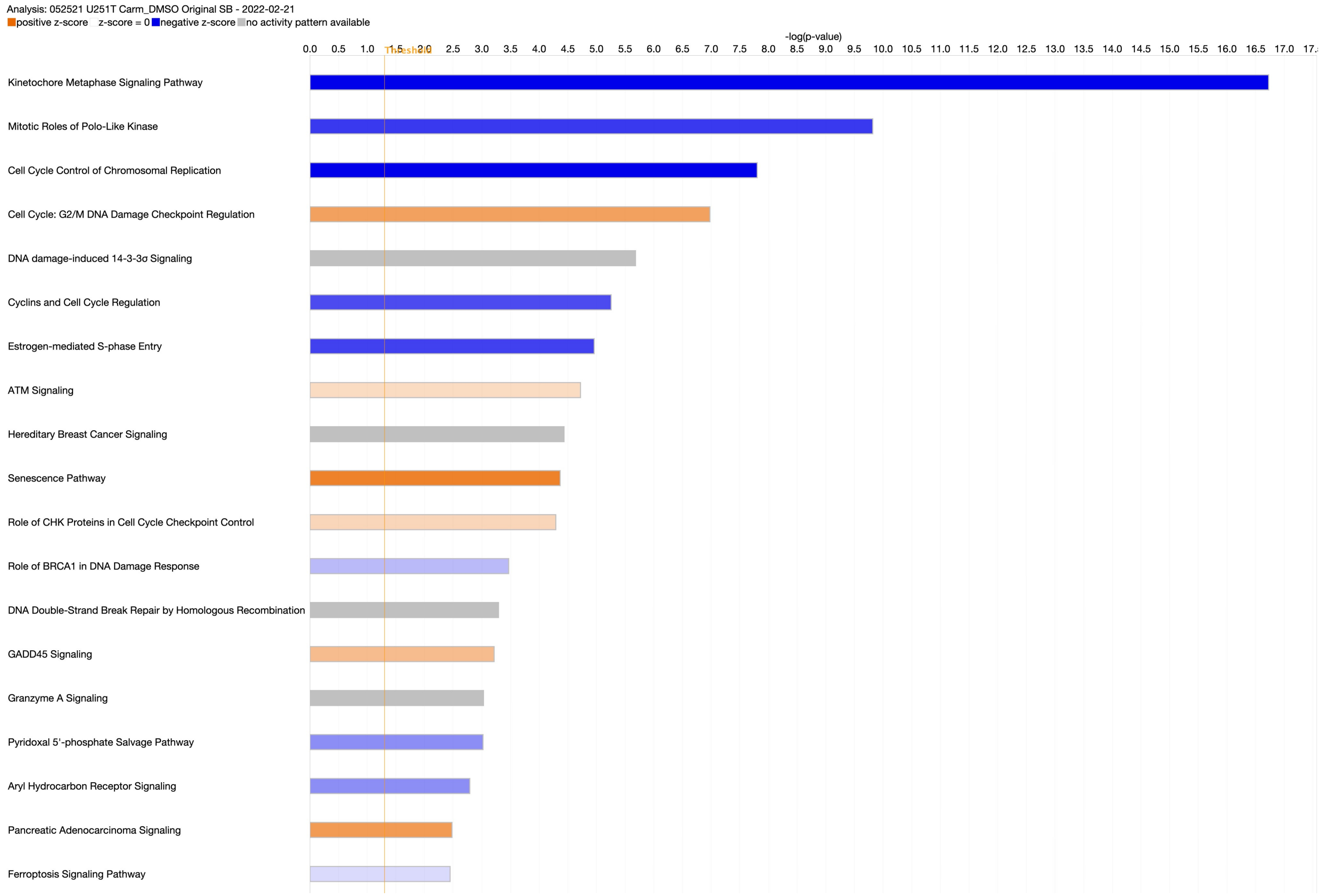

Analysis: 052521 U251T Carm_DMSO Original SB - 2022-02-21
positive z-score
z-score = 0
negative z-score
no activity pattern available
-log(p-value)
0.0 0.5 1.0 1.5 2.0 2.5 3.0 3.5 4.0 4.5 5.0 5.5 6.0 6.5 7.0 7.5 8.0 8.5 9.0 9.5 10.0 10.5 11.0 11.5 12.0 12.5 13.0 13.5 14.0 14.5 15.0 15.5 16.0 16.5 17.0
Threshold
Kinetochore Metaphase Signaling Pathway
Mitotic Roles of Polo-Like Kinase
Cell Cycle Control of Chromosomal Replication
Cell Cycle: G2/M DNA Damage Checkpoint Regulation
DNA damage-induced 14-3-3σ Signaling
Cyclins and Cell Cycle Regulation
Estrogen-mediated S-phase Entry
ATM Signaling
Hereditary Breast Cancer Signaling
Senescence Pathway
Role of CHK Proteins in Cell Cycle Checkpoint Control
Role of BRCA1 in DNA Damage Response
DNA Double-Strand Break Repair by Homologous Recombination
GADD45 Signaling
Granzyme A Signaling
Pyridoxal 5'-phosphate Salvage Pathway
Aryl Hydrocarbon Receptor Signaling
Pancreatic Adenocarcinoma Signaling
Ferroptosis Signaling Pathway

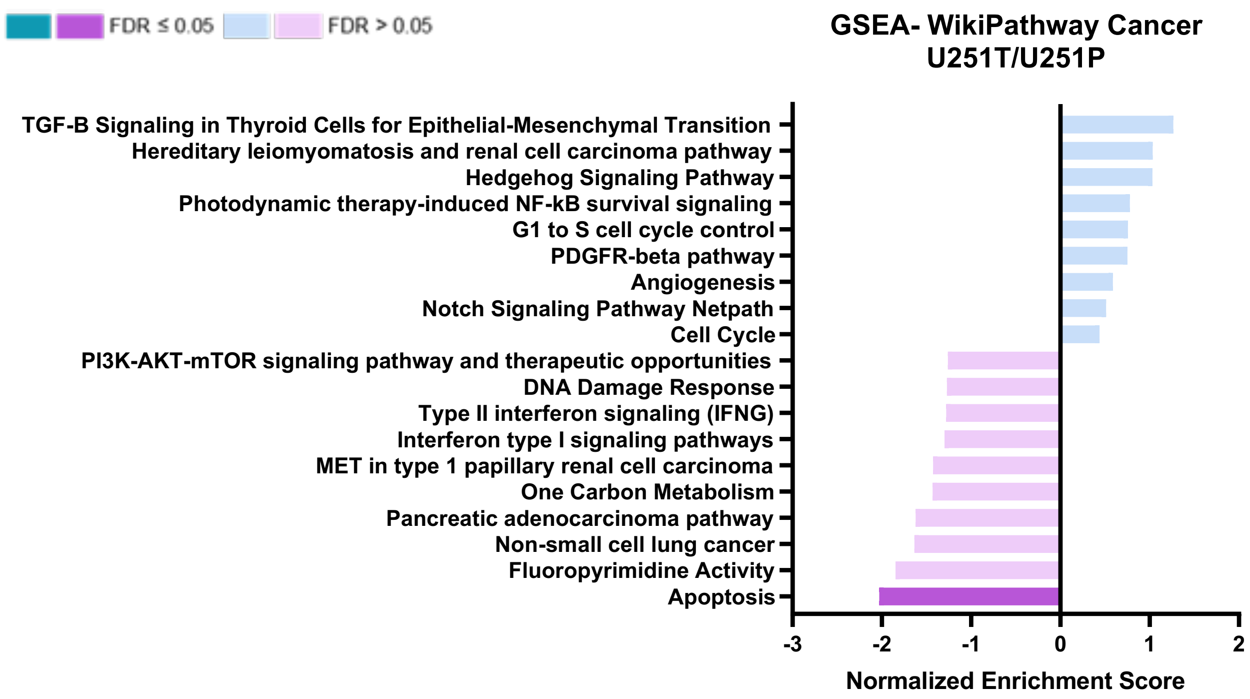
FDR ≤ 0.05
FDR > 0.05
GSEA- WikiPathway Cancer
U251T/U251P
TGF-B Signaling in Thyroid Cells for Epithelial-Mesenchymal Transition
Hereditary leiomyomatosis and renal cell carcinoma pathway
Hedgehog Signaling Pathway
Photodynamic therapy-induced NF-kB survival signaling
G1 to S cell cycle control
PDGFR-beta pathway
Angiogenesis
Notch Signaling Pathway Netpath
Cell Cycle
PI3K-AKT-mTOR signaling pathway and therapeutic opportunities
DNA Damage Response
Type II interferon signaling (IFNG)
Interferon type I signaling pathways
MET in type 1 papillary renal cell carcinoma
One Carbon Metabolism
Pancreatic adenocarcinoma pathway
Non-small cell lung cancer
Fluoropyrimidine Activity
Apoptosis
-3
-2
-1
0
1
2
Normalized Enrichment Score

Gene Set	Description	Size	Leading Edge Number	ES	NES	P Value	FDR
WP3859	TGF-B Signaling in Thyroid Cells for Epithelial-Mesenchymal Transition	10	10	0.41255	1.2649	0.2025	1
WP4206	Hereditary leiomyomatosis and renal cell carcinoma pathway	10	9	0.33391	1.0345	0.3641	1
WP4249	Hedgehog Signaling Pathway	15	2	0.29043	1.0313	0.42416	1
WP3617	Photodynamic therapy-induced NF-kB survival signaling	12	4	0.23882	0.7785	0.74877	1
WP45	G1 to S cell cycle control	29	2	0.1744	0.7561	0.79753	1
WP3972	PDGFR-beta pathway	12	6	0.23357	0.7526	0.77017	1
WP1539	Angiogenesis	12	7	0.18061	0.5879	0.9525	1
WP61	Notch Signaling Pathway Netpath	19	16	0.13555	0.5117	0.97927	1
WP179	Cell Cycle	44	2	0.09449	0.4382	1	0.99752
WP3844	PI3K-AKT-mTOR signaling pathway and therapeutic opportunities	16	9	-0.3742	-1.259	0.20202	0.59865
WP707	DNA Damage Response	28	6	-0.3268	-1.272	0.16272	0.60929
WP619	Type II interferon signaling (IFNG)	11	7	-0.4343	-1.278	0.16275	0.64054
WP585	Interferon type I signaling pathways	21	10	-0.3633	-1.298	0.16172	0.636
WP4205	MET in type 1 papillary renal cell carcinoma	23	7	-0.3851	-1.425	0.093802	0.53673
WP241	One Carbon Metabolism	12	6	-0.4681	-1.431	0.098684	0.60574
WP4263	Pancreatic adenocarcinoma pathway	37	12	-0.3826	-1.621	0.01248	0.26324
WP4255	Non-small cell lung cancer	35	13	-0.3954	-1.634	0.012559	0.32067
WP1601	Fluoropyrimidine Activity	18	13	-0.5249	-1.846	0.003373	0.07029
WP254	Apoptosis	30	16	-0.5073	-2.028	0	0.01378

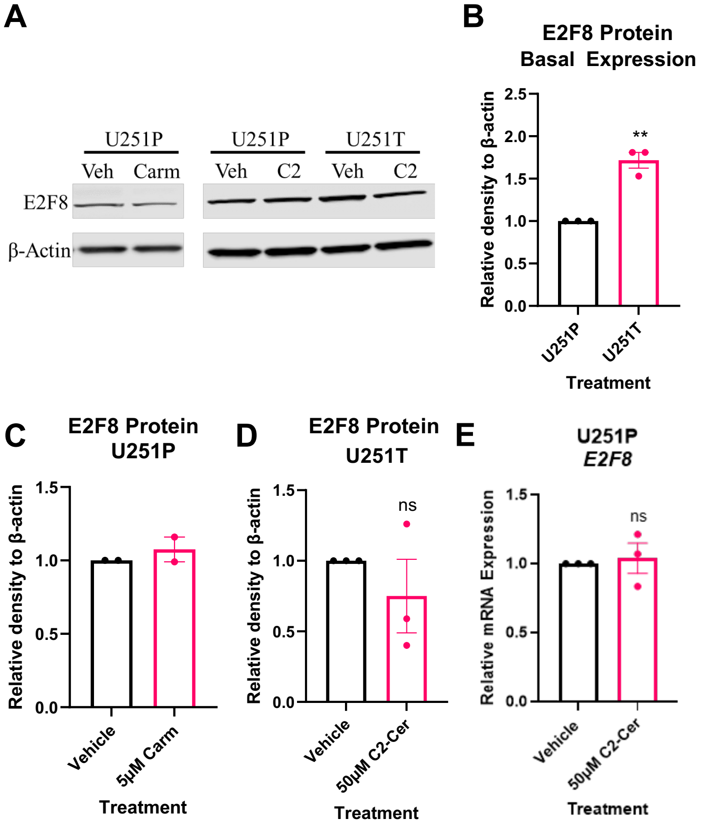
A
U251P
Veh
Carm
U251P
Veh
C2
U251T
Veh
C2
E2F8
β-Actin
B
E2F8 Protein
Basal Expression
Relative density to β-actin
**
U251P
U251T
Treatment
C
E2F8 Protein
U251P
Relative density to β-actin
Vehicle
5μM Carm
Treatment
D
E2F8 Protein
U251T
Relative density to β-actin
ns
Vehicle
50μM C2-Cer
Treatment
E
U251P
E2F8
Relative mRNA Expression
ns
Vehicle
50μM C2-Cer
Treatment

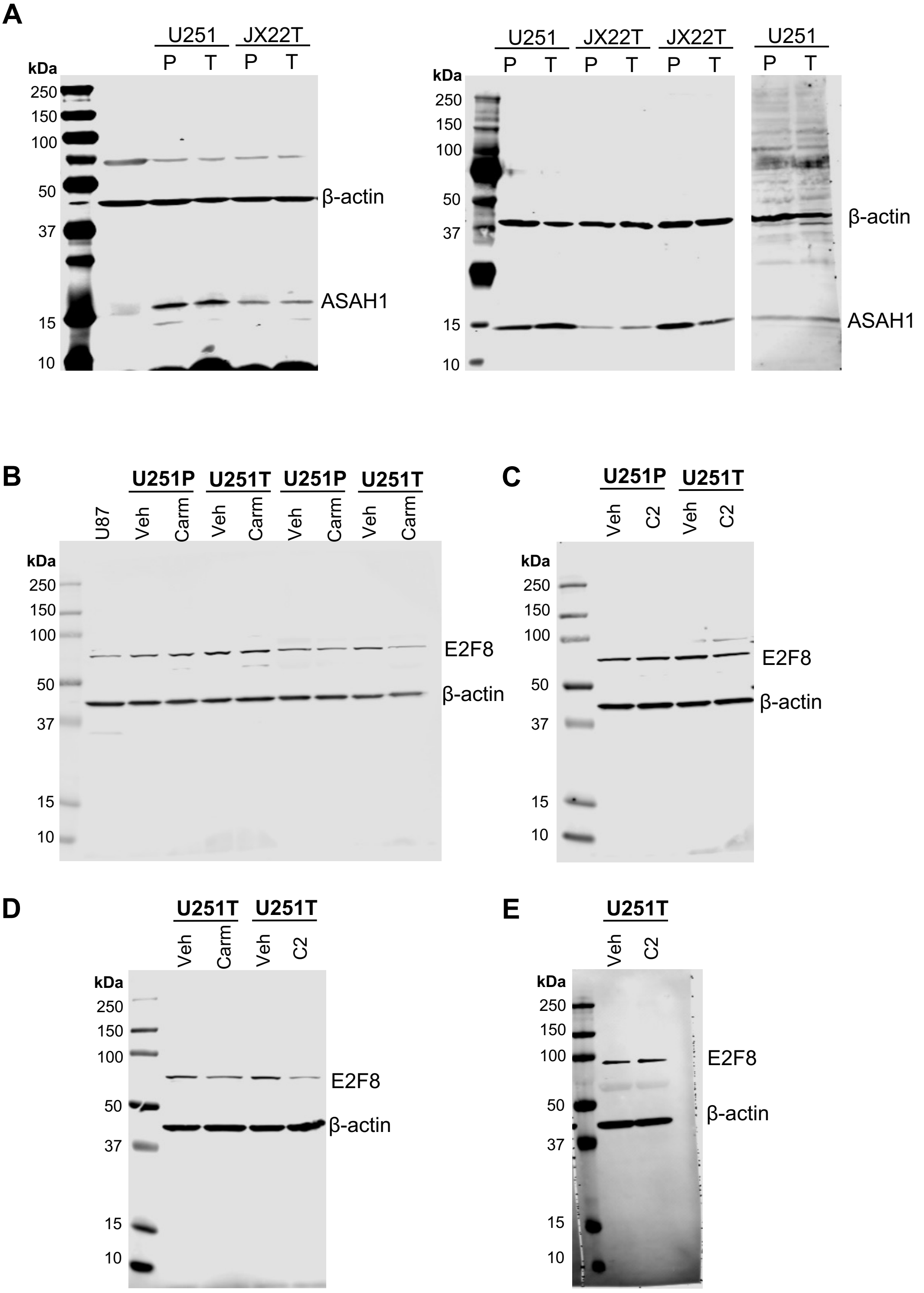
A
U251
JX22T
P
T
P
T
kDa
250
150
100
50
37
15
10
β-actin
ASAH1
U251
JX22T
JX22T
U251
B
U251P
U251T
U251P
U251T
U87
Veh
Carm
E2F8
β-actin
C
U251P
U251T
Veh
C2
D
U251T
U251T
E
U251T

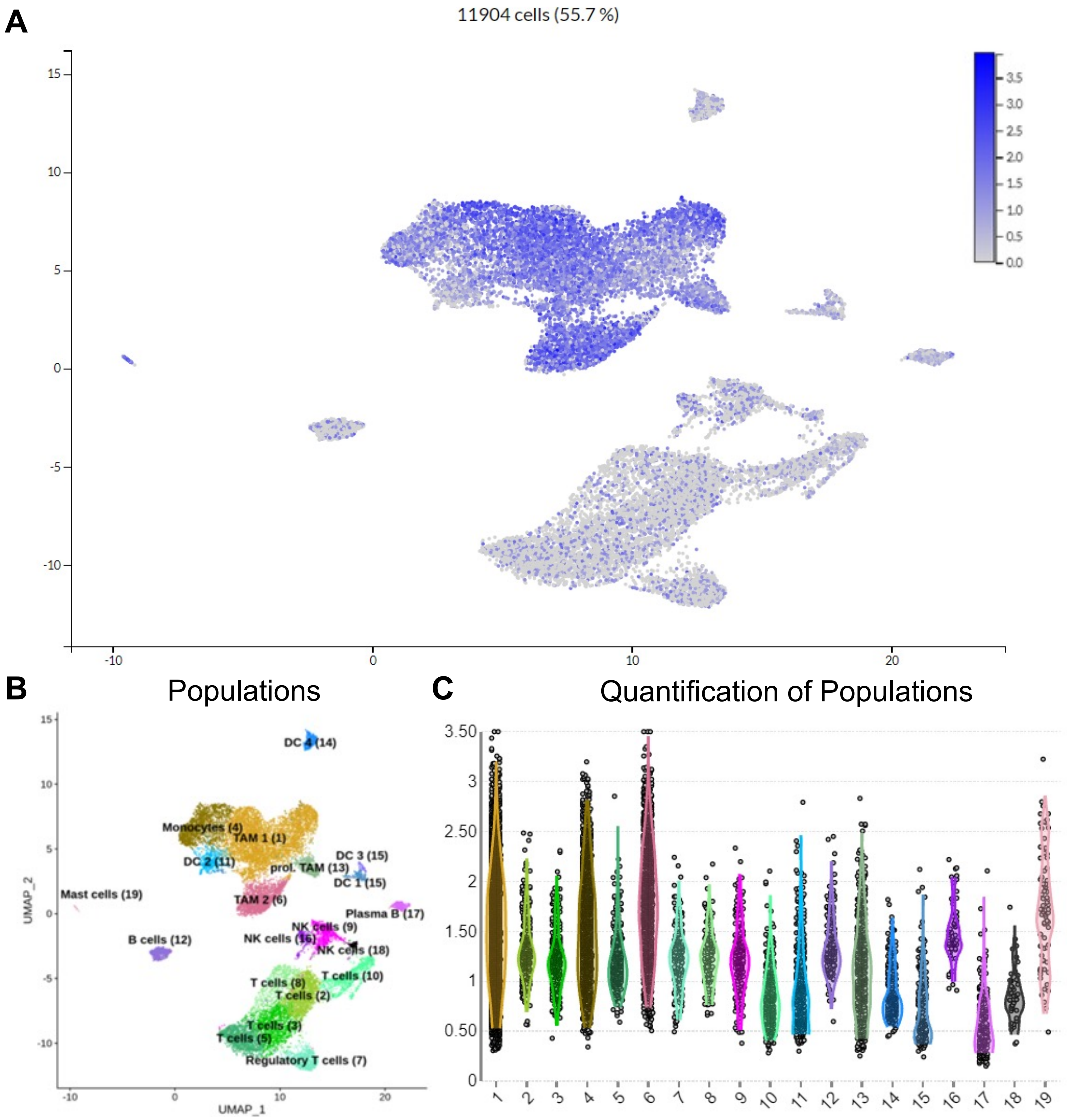
A
11904 cells (55.7 %)
B
Populations
DC 4 (14)
Monocytes (4)
TAM 1 (1)
DC 2 (11)
prol. TAM (13)
DC 3 (15)
DC 1 (15)
Mast cells (19)
TAM 2 (6)
Plasma B (17)
NK cells (9)
B cells (12)
NK cells (16)
NK cells (18)
T cells (8)
T cells (10)
T cells (2)
T cells (3)
T cells (5)
Regulatory T cells (7)
UMAP_1
UMAP_2
C
Quantification of Populations
